# Influence of temperature and precipitation on dengue incidence in Campinas, São Paulo State, Brazil (2013-2022)

**DOI:** 10.1590/0037-8682-0080-2024

**Published:** 2024-09-02

**Authors:** Bernardo Geraldini, Igor Cavallini Johansen, Marcelo Justus

**Affiliations:** 1 Universidade Estadual de Campinas, Instituto de Economia, Campinas, SP, Brasil.; 2 Universidade Estadual de Campinas, Instituto de Filosofia e Ciências Humanas, Departamento de Demografia, Campinas, SP, Brasil.; 3 Universidade Estadual de Campinas, Instituto de Economia, Centro de Estudos em Economia Aplicada, Agrícola e do Meio Ambiente, Campinas, SP, Brasil.

**Keywords:** Dengue, Time series, Climate, Temperature, Precipitation

## Abstract

**Background:**

Global dengue cases are rising, notably in Brazil.

**Methods:**

By using monthly data, we estimated linear regressions with ARIMA errors to measure the influence of temperature and precipitation on dengue incidence in the city of Campinas, São Paulo State, Brazil.

**Results:**

Findings suggest that a 1°C increase in mean temperature can lead to a cumulative increase of up to 40% in dengue incidence within 2 months. Precipitation shows no significant impact.

**Conclusions:**

Results highlight the importance of temperature on the spread of dengue and potentially other mosquito-borne diseases.

Global dengue incidence has increased over the past years. A recent report by the World Health Organization (WHO) points to a ten-fold increase in reported cases from 2000 to 2019, with more than five million cases registered in 2019^1^. Brazil has been particularly affected and recorded more than 1.5 million cases in 2023, a surge of > 65% compared to 2 years prior^2^.

Climate change is set to modify the scenario of infectious diseases, particularly mosquito-borne illnesses like dengue, yellow fever, Chikungunya, and Zika^3^. Although increases in temperature (up to 30°C) and precipitation are commonly found to be associated with increased dengue incidence, recent studies have shown that general explanations concerning climate are not capable of explaining the dynamics of the disease^4^ ^,^ ^5^ ^,^ ^6^. Hence, the interconnections between mosquito vectors, the environment, and disease transmission pose a significant challenge for precise forecasting, which is crucial for public health readiness.

Since urban and climate specificities directly shape dengue incidence, we investigated the influence of precipitation and temperature on the dengue spread in the city of Campinas, São Paulo State, Brazil. Similar to Brunkard *et al.* (2008)^7^ and Gharbi *et al.* (2011)^8^, we estimated linear regressions with autoregressive integrated moving average (ARIMA) errors. Both precipitation and temperature were included as independent variables, whereas dengue incidence per 100,000 population served as the dependent variable.

Monthly number of dengue cases was obtained from the State Health Department^9^. To smooth the series, we followed a procedure similar to the one employed by Martinez et al. (2011)^10^: a value of 1 was added to all observations to allow for logarithmic transformation of the series. Annual population count was obtained from the Brazilian Institute of Geography and Statistics (IBGE)^11^ and interpolated linearly to provide monthly estimates. Temperature and precipitation data were obtained from the Center for Meteorological and Climatic Research Applied to Agriculture (CEPAGRI)^12^. Climate variables were also logarithmized. Data covers the period from January 2013 to December 2022^§^. Stationarity is a key requirement when estimating time series models since an underlying assumption is that the time series data shows a stable statistical structure over time. Stationarity was verified using the Kwiatkowski-Phillips-Schmidt-Shin (KPSS) test. Model specification was performed automatically with *fable* package for *R* ^13^. The selection process for the seasonal and non-seasonal ARIMA models was carried out automatically, aiming to minimize the Akaike Information Criterion. As a measure of regression performance, we provide the standardized root mean square error (SRMSE), obtained by dividing the model's root mean square error (RMSE) by the standard deviation of the series of cases. An SRMSE > 1 indicates that predictions are less accurate than assuming the mean of the series^14^. Complete modeling information, including ARIMA coefficients and analysis of residuals, as well as the *R* code used, is available in the supplementary material. Time dummies were introduced to account for the three months where dengue incidence was > 1,000 per 100,000 population. This adjustment was implemented to capture and accommodate the unique temporal patterns associated with these particular periods better. 

Figure 1 displays the logarithm of the monthly observations for the three analyzed series in the city of Campinas: the top panel shows the number of dengue cases (plus one) per 100,000 population; the middle panel shows the mean temperature; and the lower panel shows precipitation. Seasonality is evident in each series, as confirmed by the autocorrelation function provided in the supplementary material.

**FIGURE 1:**
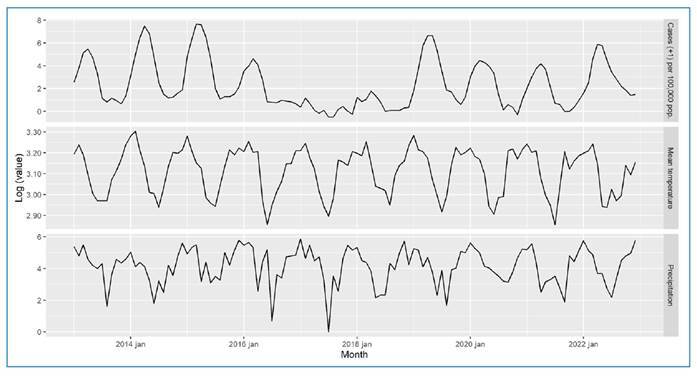
Logarithm of monthly observations for the modeled series in the city of Campinas (2013 - 2022). Top panel: number of dengue cases (plus one) per 100,000 population. Middle panel: mean temperature. Lower panel: precipitation.

Following Hyndman’s^15^ notation, basic model specification is

$$y_{t}=\;\beta_{0}+\beta_{1}x_{t-i}+\eta_{t}$$

where 𝑦_𝑡_ is the logarithm of the number of dengue cases (plus one) per 100,000 population at time 𝑡, 𝛽_1_ is the vector of estimated coefficients, 𝑥_𝑡−𝑖_ is a vector of the exogenous variables (precipitation and temperature) at time 𝑡−𝑖 (where 0 ≤ 𝑖 ≤ 2), and the error term 𝜂_𝑡_ is modeled using 𝐴𝑅𝐼𝑀𝐴 (𝑝,𝑑,𝑞)(𝑃,𝐷,𝑄)𝑠, that is, accounting for seasonality.

In our modeling strategy, we first proceeded by estimating a pure seasonal 𝐴𝑅𝐼𝑀𝐴 model - *i.e.*, a model without exogenous variables. As expected and shown in Figure 1, dengue incidence exhibits a highly seasonal pattern. The model automatically selected was 𝐴𝑅𝐼𝑀𝐴 (2,0,0)(2,1,0)12, as detailed in Table 1. However, by incorporating temperature and precipitation as exogenous variables, seasonality is almost entirely accounted for. Intermediate models (Models 2 to 4), which include an increasing number of lags for the climate variables, demonstrate a reduction in the number of seasonal coefficients. In the selected models (Models 5 and 6), no seasonal coefficients are present. This absence indicates that seasonality is effectively captured by the climate variables and by the dummy variable.

**TABLE 1:** Estimation results from the seasonal ARIMA model (Model 1) and regression models with ARIMA errors (Models 2-6).

Exogenous variables	Regression with ARIMA errors					
	Model 1:	Model 2:	Model 3:	Model 4:	Model 5:	Model 6:
	ARIMA	ARIMA	ARIMA	ARIMA	ARIMA	ARIMA
	(2,0,0)(2,1,0)_12_	(2,0,0)(2,1,0)_12_	(0,0,5)(1,0,0)_12_	(2,0,0)(1,0,0)_12_	(3,0,3)	(2,0,4)
Temperature (*t*)	-	NS	NS	2.82*** (1.03)	3.13*** (0.91)	4.27*** (0.91)
Temperature (*t-*1)	-	-	NS	2.25** (1.05)	2.13** (0.88)	2.61*** (0.90)
Temperature (*t-*2)	-	-	-	1.79* (1.04)	-	2.56*** (0.94)
Precipitation (*t*)	-	NS	NS	NS	NS	NS
Precipitation (*t-*1)	-	-	NS	NS	NS	NS
Precipitation (*t-*2)	-	-	-	NS	-	NS
Dummy	No	No	No	No	Yes	Yes
**Diagnostics tests and other measures**						
Shapiro-Wilk test	*p* < 0.01	*p* < 0.01	*p* = 0.05	*p* = 0.67	*p* = 0.10	*p* = 0.47
ARCH-LM test	*p* = 0.24	*p* = 0.27	*p* = 0.57	*p* = 0.88	*p* = 0.84	*p* = 0.68
Ljung-Box test: *Q*(18)	*p* < 0.01	*p* < 0.01	*p* < 0.01	*p* = 0.01	*p* = 0.29	*p* = 0.22
*Q*(24)	*p* < 0.01	*p* < 0.01	*p =* 0.03	*p =* 0.03	*p =* 0.57	*p =* 0.50
AIC	225.50	227.88	245.61	245.53	241.22	238.44
SRMSE	*-*	*-*	*-*	*-*	0.4125	0.4108

**Notes:** *, **, and *** denote statistical significance of at least 10%, 5%, and 1%, respectively. The values between parentheses indicate the standard error of the coefficient. NS denotes ‘not significant at the 5% level’. Sample size is 120.

Regarding the exogenous variables, we modeled two lag specifications. In the first specification, temperature and rain have an impact on dengue incidence within the same month, and an impact lagged by 1 month - *i.e.*, dengue incidence at month 𝑡 is affected by these climate variables at month 𝑡 and at month 𝑡−1. In the second specification, dengue incidence is affected by climate variables at months 𝑡, 𝑡−1, and 𝑡−2. Additionally, models with three lags were estimated but yielded insignificant coefficients for the third lag of the climate variables and were therefore excluded. These models are available in the supplementary material.

Table 1 presents the regression results, with selected models in boldface. Diagnostic tests guided model selection and are listed at the bottom of the table. 

In the selected models (Models 5 and 6), since all variables are logarithmized, coefficients represent the elasticity of dengue incidence concerning temperature and precipitation. In Model 5, for example, a 1% rise in temperature leads to a 3.1% increase in dengue incidence within the same month. Assuming a mean temperature of 25°C would mean that a 1°C rise (or 4% of the initial temperature) leads to a 12.4% increase in dengue incidence within the same month and an 8.4% surge in the next month. Hence, Model 5 suggests that a 1°C rise in temperature results in a combined increase in dengue incidence of approximately 20%. 

In Model 6, a 1°C rise in temperature leads to a 16.8% increase within the same month, 10.4% in one month, and 10% in two months - *i.e.*, the total increase in dengue incidence could reach almost 40% after two months.

Precipitation played no statistically significant role in predicting dengue incidence in Campinas, although two observations must be made. First, the model assumes a linear relationship between the climate variables and dengue incidence, and non-linearities may be present - for instance, given the municipality’s urban and sociodemographic characteristics, a minimal amount of precipitation may be necessary to allow for mosquito reproduction, whereas heavy precipitation may eliminate breeding habitats^16^. It is possible that our model failed to capture such a non-linear relationship. Second, different lag effects (such as weekly or biweekly effects) could be pertinent^7^ and were not considered in this research. 

Breeding habitats in Campinas are mostly containers such as plant pots, animal waterers, dismountable swimming pools, cans, bottles, and buckets, among others^17^. The abundance of such containers directly stems from human behavior and does not solely rely on rainwater for filling. Moreover, the impact of precipitation can occur indirectly. For example, during the 2014 epidemic in Campinas, which coincided with a severe drought^18^, part of the population began storing water in barrels at home, often without proper covering, thus facilitating the proliferation of breeding sites.

This study has some limitations. Dengue is a complex disease influenced by multiple factors, requiring a comprehensive understanding of the various elements that collectively contribute to triggering or preventing epidemics. Models like the one presented here assume that factors influencing disease incidence are stable. Such an assumption is invalid, for example, if a new virus serotype is introduced to a naive community. Moreover, urban and spatial characteristics (known to impact dengue incidence in Campinas^19^) were not considered due to the nature of the model. 

Another limitation is that dengue case reporting accuracy has improved over time, yet it remains reliant on secondary data provided by the Campinas Health Department via the reporting system. Such reliance on secondary data is a constraint inherent to long-term studies on dengue in Brazil. In Campinas, dengue notification is mandatory, following the protocols established by the Brazilian Ministry of Health^20^ and the São Paulo State Health Secretary^21^. Suspected dengue cases can be confirmed by laboratory criteria or by clinical-epidemiological linkage^§§^. However, underreporting remains a significant concern, particularly as it seems to have increased in 2020 due to the COVID-19 pandemic^22^. Additionally, a portion of the cases reported in Campinas originate from neighboring municipalities, which is another factor of uncertainty. Nonetheless, given the recent study period selected, the data available were the most appropriate and comprehensive for our investigation. 

While our focus was not to address all dengue-associated conditioning factors, we aimed to employ a promising methodology to underscore its importance and potential for predicting this disease, as well as other vector-borne illnesses, particularly in the context of a changing climate. Econometric models can serve as valuable tools to assist stakeholders in comprehending the evolving patterns of disease occurrence and formulating proactive public policies to mitigate new outbreaks.

This paper builds on a previous study published in this Journal^10^, which predicted dengue cases in Campinas using a SARIMA model. We were able to complement the previous analysis by incorporating two additional climate variables - temperature and precipitation - using a similar methodology, although not designed to forecast dengue incidence. Given Campinas’ location in a tropical climate zone, the possibility that rising temperatures could impact dengue incidence, as suggested by our models, is alarming. Brazil, as a whole, being a tropical country, faces this challenge. Despite the approval of a dengue vaccine, available in the Universal Health System since 2024, it is still limited to a very targeted population group (10-14 years old) and to only 521 out of the total 5,570 cities^23^. As such, the dengue vaccine is expected to have only marginal epidemiologic impacts over the next few years. 

Therefore, the findings of this paper remain crucial for planning surveillance and preparedness strategies. If temperature increases can exacerbate dengue incidence in areas already characterized by hot and humid tropical climates, this suggests that dengue fever may expand into cooler regions expected to warm up due to climate change, and outbreaks may intensify in already high-risk areas. Similar trends are projected for diseases such as Zika^24^ and Chikungunya^25^ in Brazil.

## Data Availability

Data and R code are available at: REDU https://doi.org/10.25824/redu/NCZHR3

## Footnotes

§ Epidemiological data for dengue in Campinas are available from 1998 onwards. However, data from 1998 to 2012 were removed from this analysis since ARIMA models estimated with the full sample showed poor fit and persistent autocorrelation (even though temperature coefficients were similar to the ones reported here). This suggests that intervention and/or transfer function analysis should be considered (in addition to multivariate models) when analyzing the full sample.

§§ The laboratory confirmation criteria include the following tests and their respective results: a. Detection of reactive NS1 protein; b. Positive viral isolation; c. Detectable RT-PCR (up to the fifth day after the onset of symptoms); d. Detection of IgM antibodies by ELISA (from the sixth day after the onset of symptoms); e. A ≥4-fold increase in antibody titers in PRNT or IH test, using paired samples (acute and convalescent phases with at least a 14-day interval). If specific laboratory confirmation is not possible or if laboratory results are inconclusive, confirmation by epidemiological linkage should be considered. This involves evaluating the spatial distribution of confirmed cases and the likelihood that the patient was infected based on nearby confirmed dengue cases.
