# Supplementary material for: Influence of temperature and precipitation on dengue incidence in Campinas, São Paulo State, Brazil (2013-2022)
Source: Rev Soc Bras Med Trop. 2024 Sep 2;57:e00710-2024. doi: 10.1590/0037-8682-0080-2024 (PMC11374123; doi:10.1590/0037-8682-0080-2024)
Supplement: Supplementary file 1 [file 1678-9849-rsbmt-57-e00710-2024-supp1.pdf]

```
library(readxl)
library(tidyverse)
```

```
## -- Attaching core tidyverse packages ------------------------ tidyverse 2.0.0 --
## v dplyr     1.1.4     v readr     2.1.5
## v forcats   1.0.0     v stringr   1.5.1
## v ggplot2   3.5.1     v tibble    3.2.1
## v lubridate 1.9.3     v tidyr     1.3.1
## v purrr     1.0.2
## -- Conflicts ------------------------------------------ tidyverse_conflicts() --
## x dplyr::filter() masks stats::filter()
## x dplyr::lag()    masks stats::lag()
## i Use the conflicted package (<http://conflicted.r-lib.org/>) to force all conflicts to become error
```

```
library(fpp3)
```

```
## -- Attaching packages ---------------------------------------------- fpp3 0.5 --
## v tsibble     1.1.4     v fable       0.3.4
## v tsibbledata 0.4.1     v fabletools  0.4.2
## v feasts      0.3.2
## -- Conflicts ------------------------------------------------- fpp3_conflicts --
## x lubridate::date()    masks base::date()
## x dplyr::filter()      masks stats::filter()
## x tsibble::intersect() masks base::intersect()
## x tsibble::interval()  masks lubridate::interval()
## x dplyr::lag()         masks stats::lag()
## x tsibble::setdiff()   masks base::setdiff()
## x tsibble::union()     masks base::union()
```

```
library(tsibble)
library(ggplot2)
```

```
base_dengue <- read_excel("dengue_dataset.xlsx")

### tsibble
base_dengue <- base_dengue  %>%
  mutate(Month = as.Date(
    paste(ano, mes, "01", sep = "-"),
    format = "%Y-%m-%d")) %>%
  select(-ano, -mes) %>%
  mutate(Month = yearmonth(Month)) %>%
  as_tsibble(index= Month)

# removing pre-2013 data and creating dummy

base_dengue_pos2013 <- base_dengue[181:300,]

base_dengue_pos2013_correc <- base_dengue_pos2013 %>% mutate(D1000 = 0)

base_dengue_pos2013_correc <- base_dengue_pos2013_correc %>%
  mutate(D1000 = ifelse(casosmais1_por_100k >= 1000, 1, D1000))

rm(base_dengue, base_dengue_pos2013)
```

```
# acf

# dengue cases
base_dengue_pos2013_correc |>
  gg_tsdisplay(log(casosmais1_por_100k),
               plot_type='partial', lag=24) +
  labs(title="Log (dengue cases plus one per 100,000 population)", y="")
```

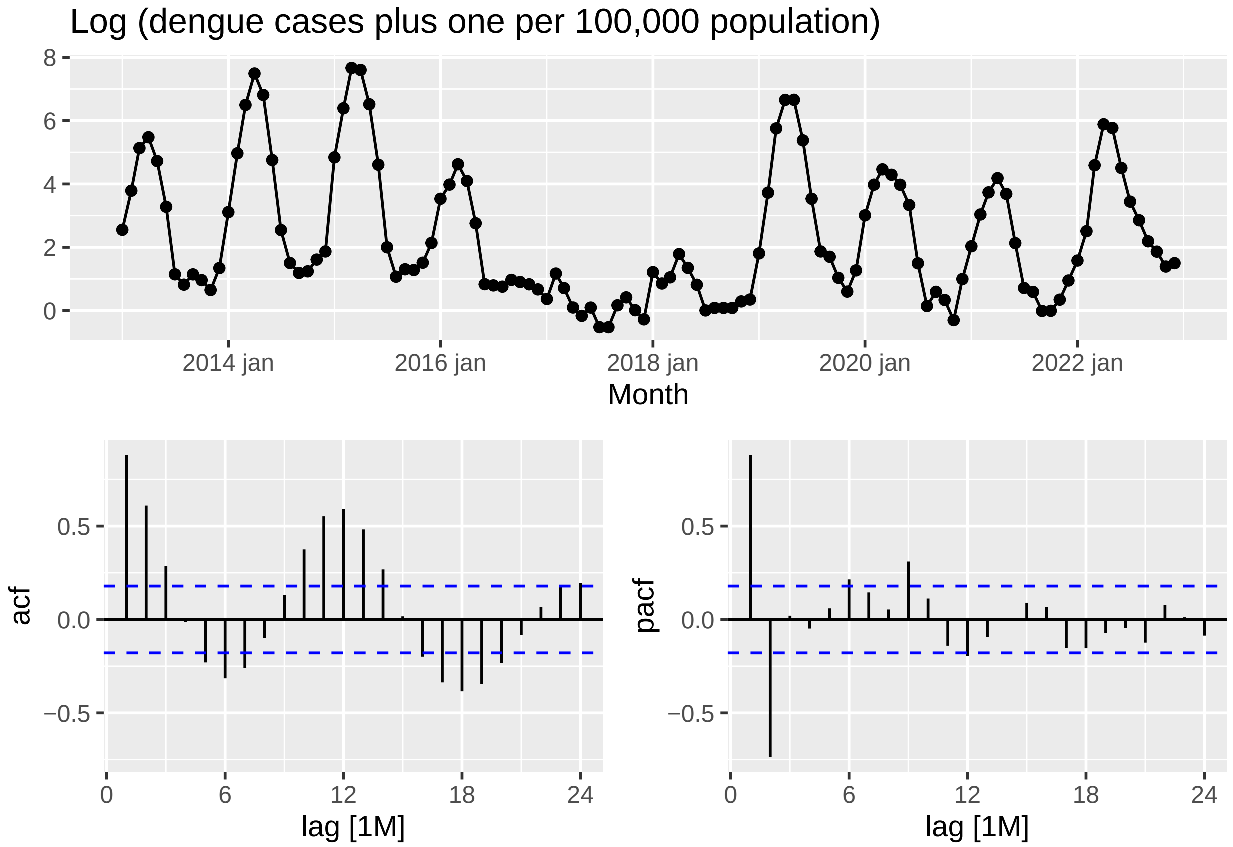


```
# temperature
base_dengue_pos2013_correc |>
  gg_tsdisplay(log(tmed),
               plot_type='partial', lag=24) +
  labs(title="Log (temperature)", y="")
```

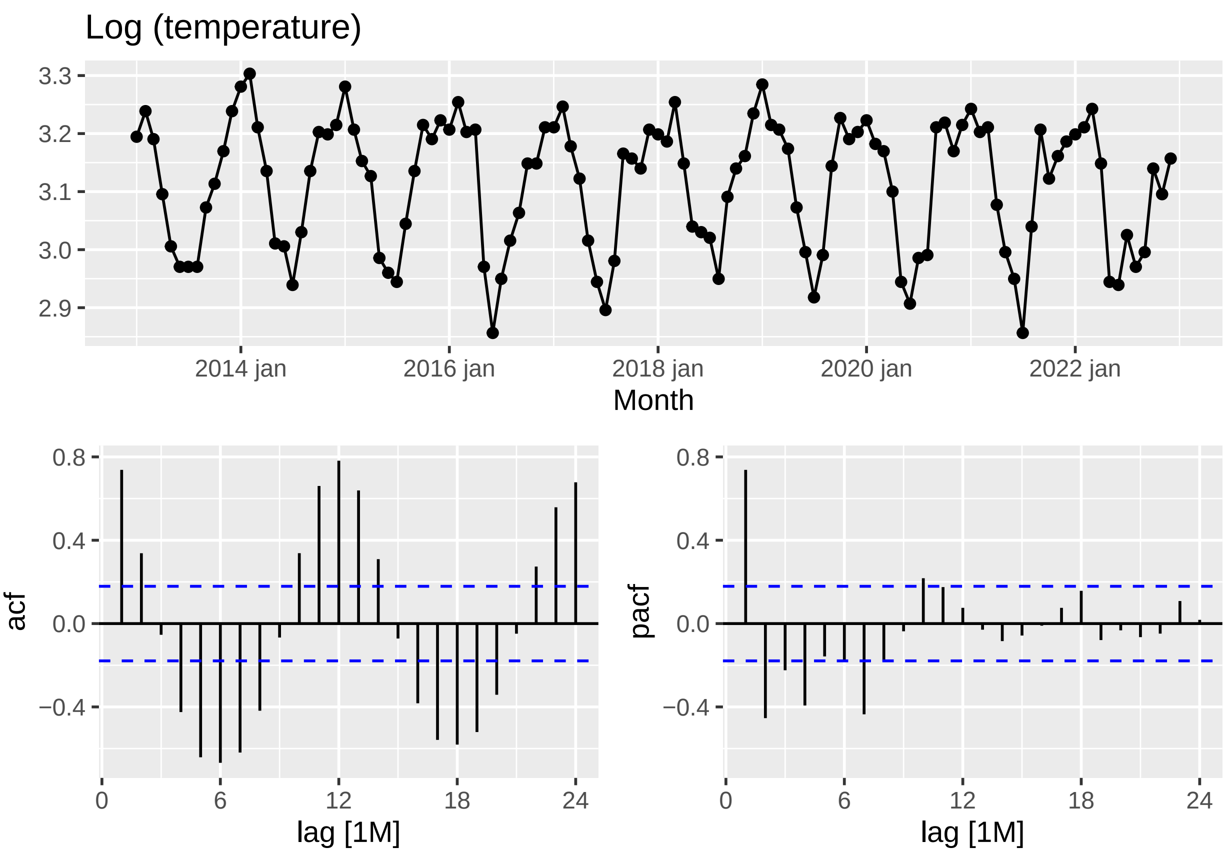


```
# precipitation
base_dengue_pos2013_correc |>
  gg_tsdisplay(log(chuva),
               plot_type='partial', lag=24) +
  labs(title="Log (precipitation)", y="")
```

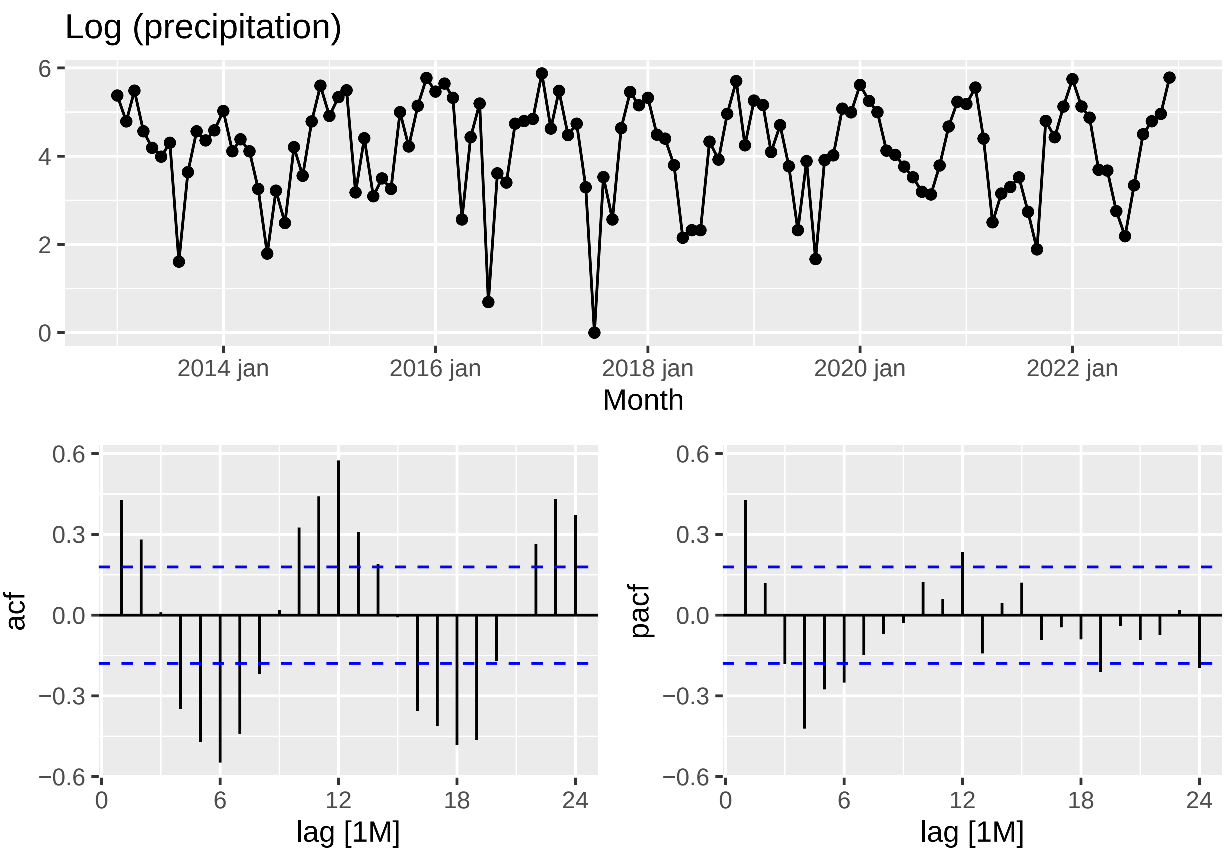


```r
# plotting logarithm of series

base_graf <- base_dengue_pos2013_correc

base_graf <- base_graf %>% rename('Cases (+1) per 100,000 pop.' = casosmais1_por_100k,
                                  'Mean temperature' = tmed,
                                  'Precipitation' = chuva)



base_graf %>%
  pivot_longer(c(`Cases (+1) per 100,000 pop.`, `Mean temperature`, Precipitation), names_to = "var", va
  mutate(value = ifelse(value > 0, log(value), NA)) %>%
  ggplot(aes(x = Month, y = value)) +
  geom_line() +
  facet_grid(vars(var), scales = "free_y") +
  scale_y_continuous(labels = scales::comma) +
  labs(title = "",
       y = "Log (value)", x = "Month")
```

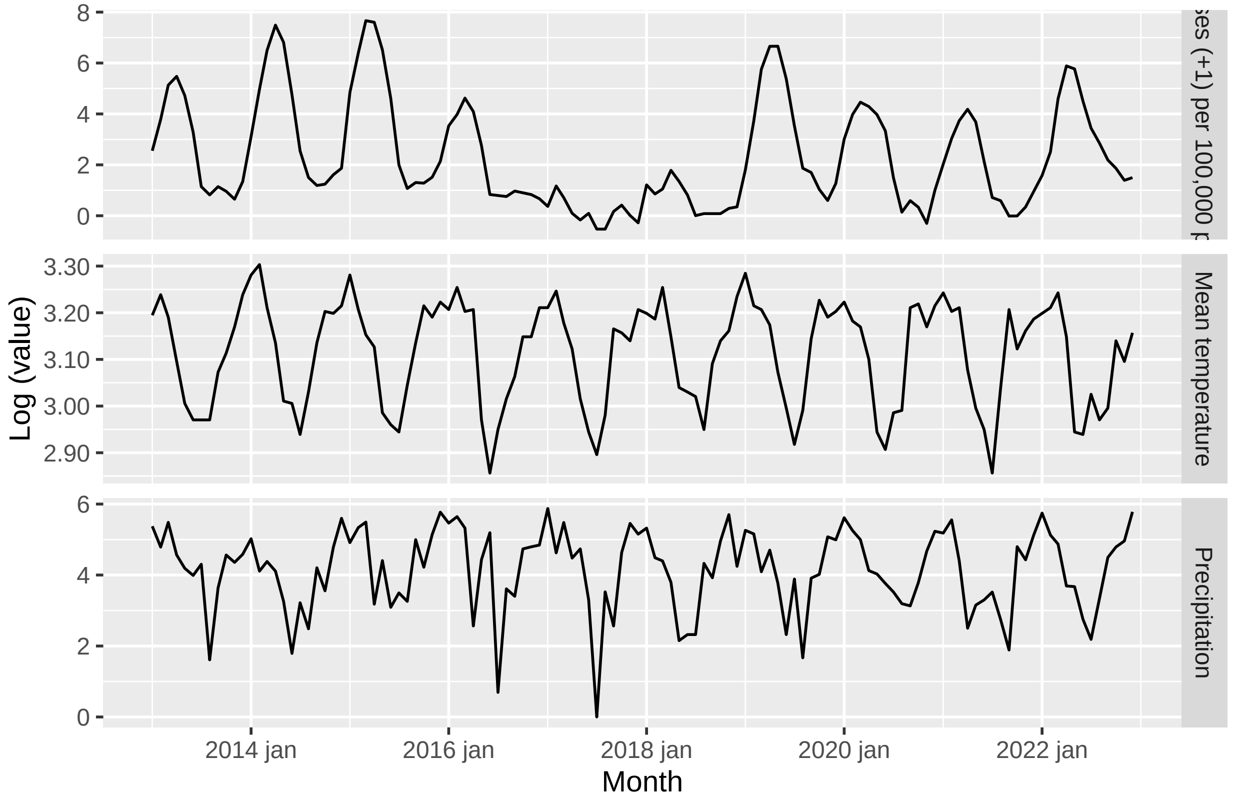

model 1

```r
# pure ARIMA

reg_log1 = base_dengue_pos2013_correc %>%
  as_tsibble() %>%
  model(arima = ARIMA(log(casosmais1_por_100k),
                      greedy = FALSE,
                      stepwise = FALSE,
                      approximation = FALSE
  )) %>%
  report(reg_log1)
```

```
## Series: casosmais1_por_100k
## Model: ARIMA(2,0,0)(2,1,0)[12]
## Transformation: log(casosmais1_por_100k)
##
## Coefficients:
##          ar1      ar2     sar1     sar2
##       1.3337  -0.4560  -0.5736  -0.3754
## s.e.  0.0955   0.0946   0.1013   0.0908
##
## sigma^2 estimated as 0.4143:  log likelihood=-107.75
```

```
## AIC=225.5   AICc=226.08   BIC=238.91
```

```
reg_log1 |> gg_tsresiduals()
```

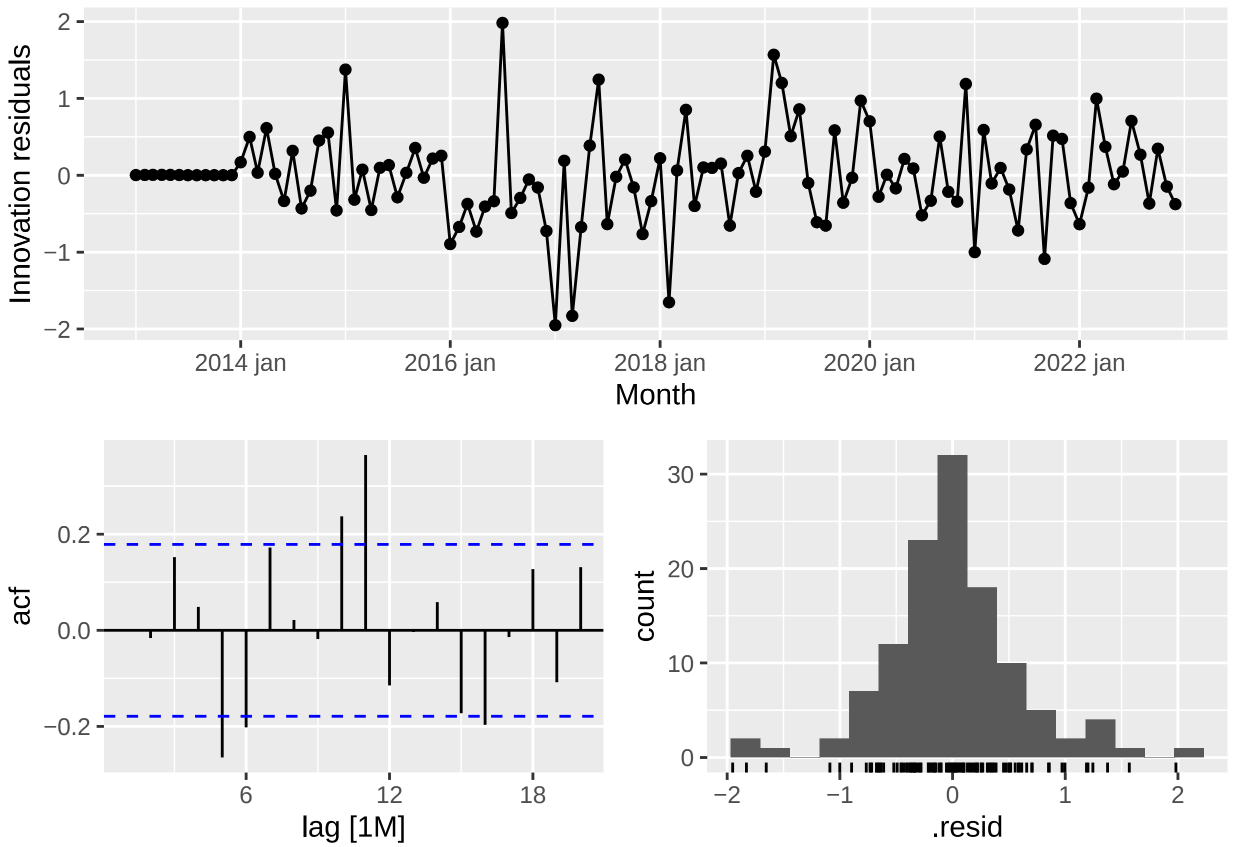


```
augment(reg_log1) |> features(.innov, ljung_box, dof=4, lag=16)
```

```
## # A tibble: 1 x 3
##   .model lb_stat    lb_pvalue
##   <chr>    <dbl>        <dbl>
## 1 arima     58.5 0.0000000423
```

```
augment(reg_log1) |> features(.innov, ljung_box, dof=4, lag=24)
```

```
## # A tibble: 1 x 3
##   .model lb_stat    lb_pvalue
##   <chr>    <dbl>        <dbl>
## 1 arima     73.2 0.0000000537
```

```
resid_model1 <- reg_log1 %>%
  residuals() %>%
  as_tibble()
```

```r
resid_model1_ts <- ts(resid_model1[3], frequency = 12, start = c(2013,1))

# heteroskedasticity

FinTS::ArchTest(resid_model1_ts)
```

```
##
##  ARCH LM-test; Null hypothesis: no ARCH effects
##
## data:  resid_model1_ts
## Chi-squared = 14.876, df = 12, p-value = 0.2483
```

```r
# normality

# Shapiro-Wilk

shapiro.test(resid_model1_ts)
```

```
##
##  Shapiro-Wilk normality test
##
## data:  resid_model1_ts
## W = 0.96238, p-value = 0.001975
```

model 2

```r
# Model 2: Controlling for contemporaneous precipitation and temperature

reg_log2 = base_dengue_pos2013_correc %>%
  as_tsibble() %>%
  model(arima = ARIMA(log(casosmais1_por_100k) ~
                      log(tmed) + log(chuva),
                      greedy = FALSE,
                      stepwise = FALSE
  )) %>%
  report(reg_log2)
```

```
## Series: casosmais1_por_100k
## Model: LM w/ ARIMA(2,0,0)(2,1,0)[12] errors
## Transformation: log(casosmais1_por_100k)
##
## Coefficients:
##          ar1      ar2      sar1     sar2   log(tmed)  log(chuva)
##       1.3436  -0.4671  -0.5701  -0.3693     0.9845     -0.0095
## s.e.  0.0957   0.0950   0.1038   0.0910     0.9320      0.0387
##
## sigma^2 estimated as 0.4166:  log likelihood=-106.94
## AIC=227.88   AICc=229   BIC=246.65
```

```
reg_log2 |> gg_tsresiduals()
```

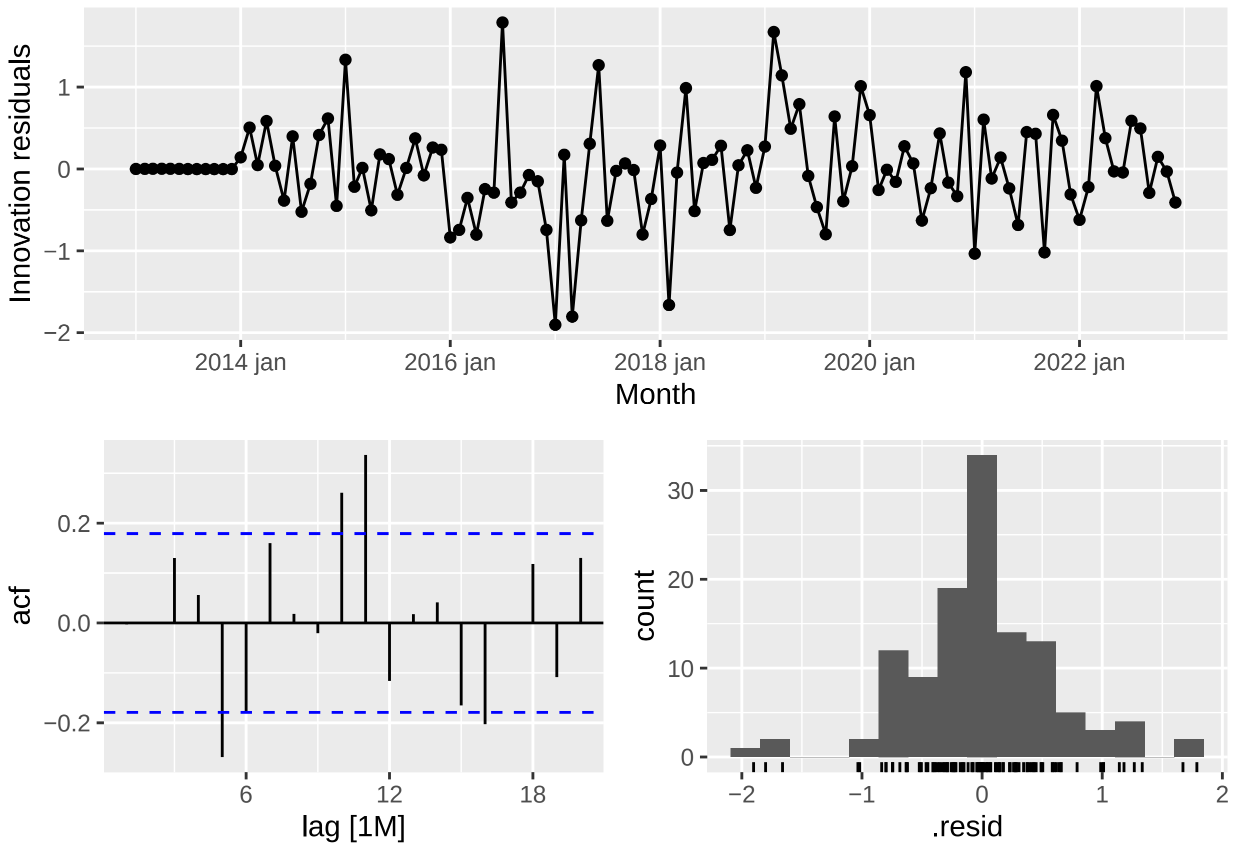


```
augment(reg_log2) |> features(.innov, ljung_box, dof=4, lag=16)
```

```
## # A tibble: 1 x 3
##   .model lb_stat   lb_pvalue
##   <chr>    <dbl>       <dbl>
## 1 arima     55.1 0.000000171
```

```
augment(reg_log2) |> features(.innov, ljung_box, dof=4, lag=24)
```

```
## # A tibble: 1 x 3
##   .model lb_stat   lb_pvalue
##   <chr>    <dbl>       <dbl>
## 1 arima     69.5 0.000000219
```

```
resid_model2 <- reg_log2 %>%
  residuals() %>%
  as_tibble()


resid_model2_ts <- ts(resid_model2[3], frequency = 12, start = c(2013,1))
```

```r
# heteroskedasticity

FinTS::ArchTest(resid_model2_ts)
```

```
##
##  ARCH LM-test; Null hypothesis: no ARCH effects
##
## data:  resid_model2_ts
## Chi-squared = 14.413, df = 12, p-value = 0.2751
```

```r
# normality

# Shapiro-Wilk

shapiro.test(resid_model2_ts)
```

```
##
##  Shapiro-Wilk normality test
##
## data:  resid_model2_ts
## W = 0.96328, p-value = 0.002341
```

model 3

```r
# Model 3: Controlling for contemporaneous precipitation and temperature, plus a one-period lag

reg_log3 = base_dengue_pos2013_correc %>%
  as_tsibble() %>%
  model(arima = ARIMA( log(casosmais1_por_100k) ~
                         log(tmed) + log(lag(tmed, 1))  +
                         log(chuva) + log(lag(chuva, 1)),
                       greedy = FALSE,
                       stepwise = FALSE,
                       approximation = FALSE
                       )) %>%
  report(reg_log3)
```

```
## Series: casosmais1_por_100k
## Model: LM w/ ARIMA(0,0,5)(1,0,0)[12] errors
## Transformation: log(casosmais1_por_100k)
##
## Coefficients:
##          ma1     ma2     ma3     ma4     ma5    sar1  log(tmed)
##       1.3813  1.4434  1.2500  0.9020  0.4922  0.3978     0.9543
## s.e.  0.0900  0.1568  0.1812  0.1434  0.1151  0.1047     0.5457
##       log(lag(tmed, 1))  log(chuva)  log(lag(chuva, 1))
##                 -0.1659      0.0057             -0.0162
## s.e.             0.5598      0.0501              0.0523
##
## sigma^2 estimated as 0.3965:  log likelihood=-111.81
## AIC=245.61   AICc=248.06   BIC=276.28
```

```
reg_log3 |> gg_tsresiduals()
```

```
## Warning: Removed 1 row containing missing values or values outside the scale range
## (`geom_line()`).

## Warning: Removed 1 row containing missing values or values outside the scale range
## (`geom_point()`).

## Warning: Removed 1 row containing non-finite outside the scale range
## (`stat_bin()`).
```

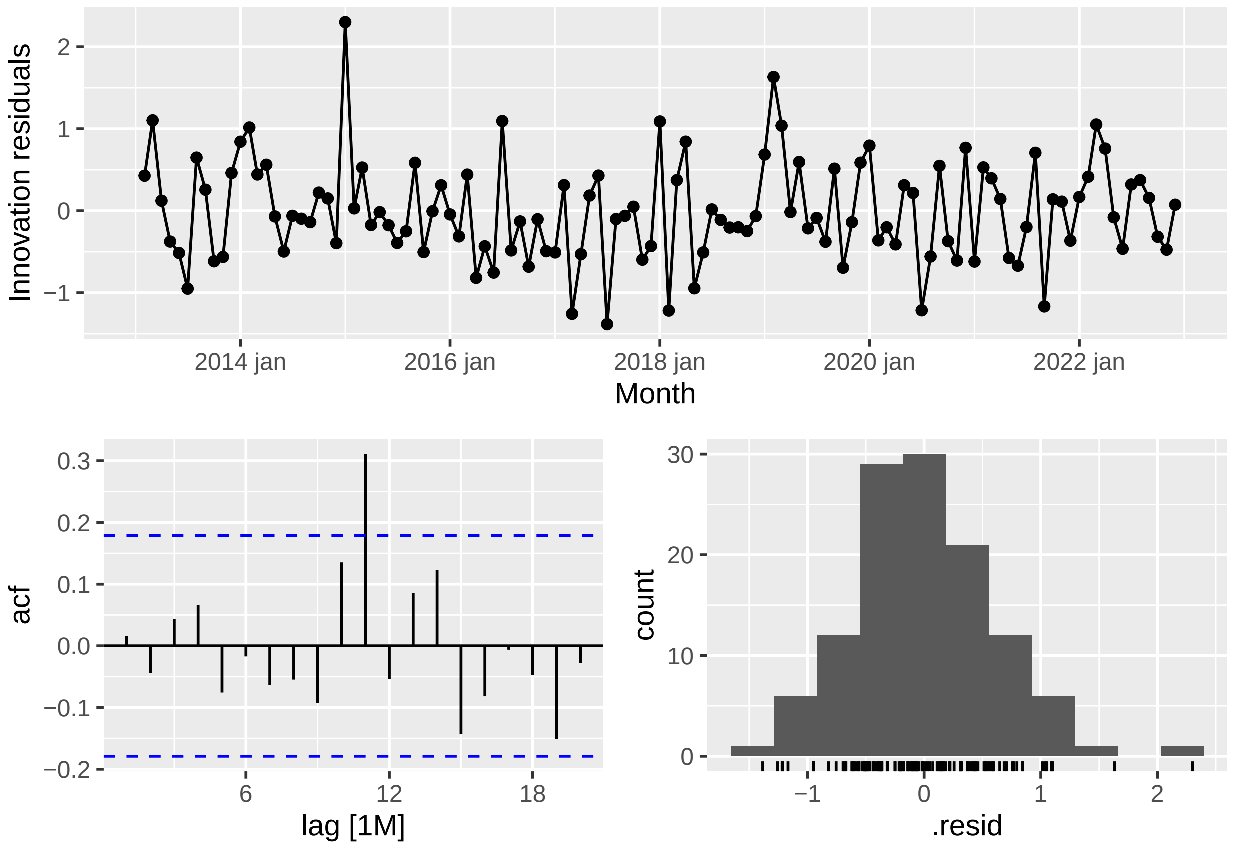


```
augment(reg_log3) |> features(.innov, ljung_box, lag=18, dof = 6)
```

```
## # A tibble: 1 x 3
##   .model lb_stat lb_pvalue
##   <chr>    <dbl>     <dbl>
## 1 arima     26.7   0.00846
```

```
augment(reg_log3) |> features(.innov, ljung_box, lag=24, dof = 6)
```

```
## # A tibble: 1 x 3
##   .model lb_stat lb_pvalue
##   <chr>    <dbl>     <dbl>
## 1 arima     30.3    0.0349
```

```
resid_model3 <- reg_log3 %>%
  residuals() %>%
  as_tibble()


resid_model3_ts <- ts(resid_model3[3], frequency = 12, start = c(2013,1))

# heteroskedasticity

FinTS::ArchTest(resid_model3_ts)
```

```
##
##  ARCH LM-test; Null hypothesis: no ARCH effects
##
## data:  resid_model3_ts
## Chi-squared = 10.477, df = 12, p-value = 0.5742
```

```
# normality

# Shapiro-Wilk

shapiro.test(resid_model3_ts)
```

```
##
##  Shapiro-Wilk normality test
##
## data:  resid_model3_ts
## W = 0.97812, p-value = 0.04932
```

model 4

```
# Model 4: Controlling for contemporaneous precipitation and temperature, plus a two-period lag

reg_log4 = base_dengue_pos2013_correc %>%
  as_tsibble() %>%
  model(arima = ARIMA( log(casosmais1_por_100k) ~
                      log(tmed) + log(lag(tmed, 1)) + log(lag(tmed, 2)) +
                      log(chuva) + log(lag(chuva, 1)) + log(lag(chuva, 2)),
                    greedy = FALSE,
                    stepwise = FALSE,
                    approximation = FALSE
                    )) %>%
  report(reg_log4)
```

```
## Series: casosmais1_por_100k
## Model: LM w/ ARIMA(2,0,0)(1,0,0)[12] errors
## Transformation: log(casosmais1_por_100k)
##
## Coefficients:
```

```
##          ar1      ar2    sar1  log(tmed)  log(lag(tmed, 1))  log(lag(tmed, 2))
##       1.4453  -0.6287  0.2531     2.8217              2.259             1.7961
## s.e.  0.0859   0.0902  0.1173     1.0391              1.051             1.0426
##       log(chuva)  log(lag(chuva, 1))  log(lag(chuva, 2))  intercept
##           0.0149              0.0588              0.0755   -19.6579
## s.e.      0.0647              0.0866              0.0645     7.7177
##
## sigma^2 estimated as 0.4058:  log likelihood=-111.77
## AIC=245.53   AICc=247.97   BIC=276.19
```

```
reg_log4 |> gg_tsresiduals()
```

```
## Warning: Removed 2 rows containing missing values or values outside the scale range
## (`geom_line()`).

## Warning: Removed 2 rows containing missing values or values outside the scale range
## (`geom_point()`).

## Warning: Removed 2 rows containing non-finite outside the scale range
## (`stat_bin()`).
```

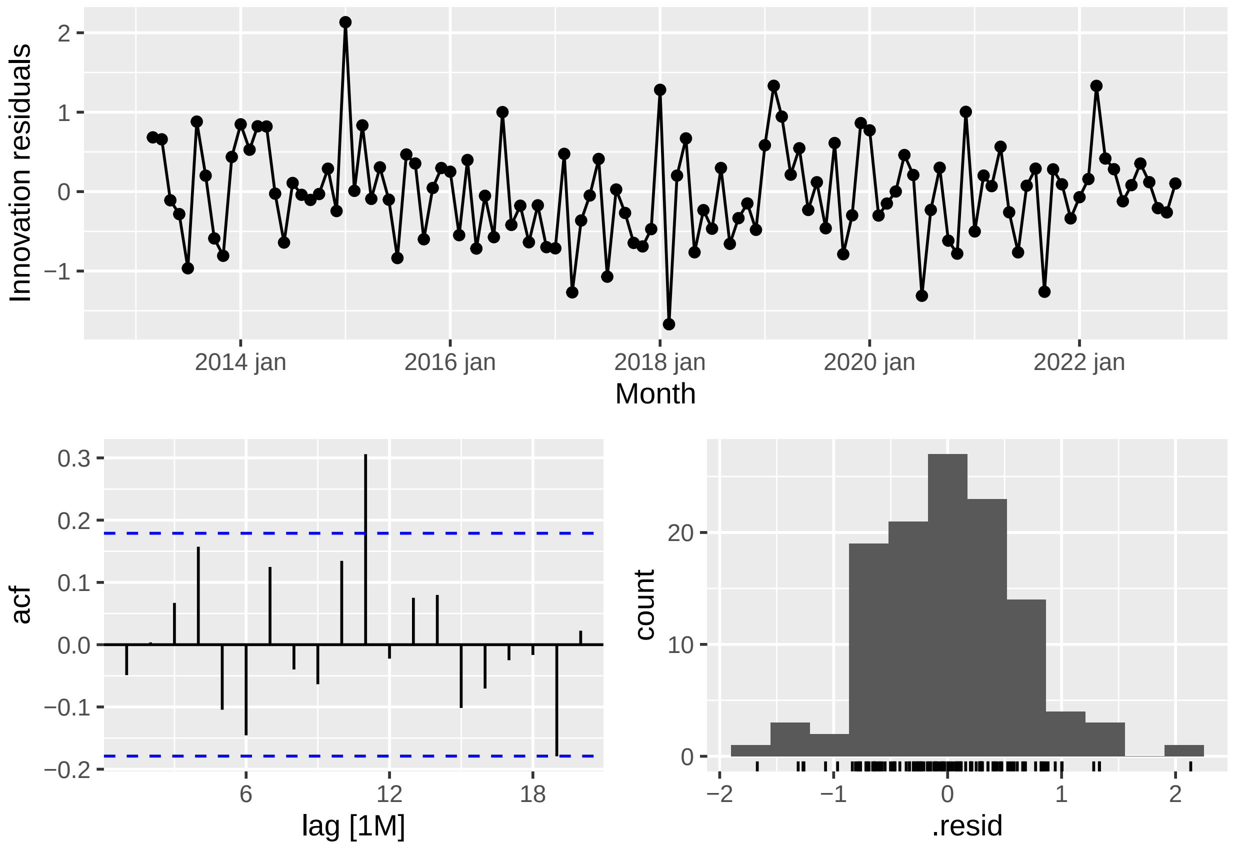


```
# report(reg_log1)

augment(reg_log4) |> features(.innov, ljung_box, lag=18, dof = 3)
```

```
## # A tibble: 1 x 3
##   .model lb_stat lb_pvalue
##   <chr>    <dbl>     <dbl>
## 1 arima     29.4    0.0144
```

```
augment(reg_log4) |> features(.innov, ljung_box, lag=24, dof = 3)
```

```
## # A tibble: 1 x 3
##   .model lb_stat lb_pvalue
##   <chr>    <dbl>     <dbl>
## 1 arima     34.5    0.0316
```

```
# ts object

resid_model4 <- reg_log4 %>%
  residuals() %>%
  as_tibble()

resid_model4_ts <- ts(resid_model4[3], frequency = 12, start = c(2013,1))

# heteroskedasticity

FinTS::ArchTest(resid_model4_ts)
```

```
##
##  ARCH LM-test; Null hypothesis: no ARCH effects
##
## data:  resid_model4_ts
## Chi-squared = 6.6384, df = 12, p-value = 0.8806
```

```
# normality

# Shapiro-Wilk

shapiro.test(resid_model4_ts)
```

```
##
##  Shapiro-Wilk normality test
##
## data:  resid_model4_ts
## W = 0.99141, p-value = 0.6764
```

model 5 (final model)

```
# Model 5 (final model): Controlling for contemporaneous precipitation and temperature, plus a one-peri

reg_log5 = base_dengue_pos2013_correc %>%
  as_tsibble() %>%
  model(arima = ARIMA( log(casosmais1_por_100k) ~
```

```
                        log(tmed) + log(lag(tmed, 1)) +
                        log(chuva) + log(lag(chuva, 1)) +
                         D1000,
                      greedy = FALSE,
                      stepwise = FALSE,
                      approximation = FALSE
                      )) %>%
  report(reg_log5)
```

```
## Warning in sqrt(diag(best$var.coef)): NaNs produzidos
```

```
## Series: casosmais1_por_100k
## Model: LM w/ ARIMA(3,0,3) errors
## Transformation: log(casosmais1_por_100k)
##
## Coefficients:
##          ar1      ar2     ar3      ma1     ma2     ma3  log(tmed)
##       2.4057  -2.1906  0.7079  -1.0481  0.2440  0.2739     3.1395
## s.e.     NaN      NaN     NaN      NaN  0.1327  0.0605     0.9141
##       log(lag(tmed, 1))  log(chuva)  log(lag(chuva, 1))   D1000  intercept
##                  2.1300      0.0098              0.0009  0.3090   -14.0952
## s.e.             0.8854      0.0552              0.0548  0.2637     4.6366
##
## sigma^2 estimated as 0.3804:  log likelihood=-107.61
## AIC=241.22   AICc=244.65   BIC=277.45
```

```
reg_log5 |> gg_tsresiduals()
```

```
## Warning: Removed 1 row containing missing values or values outside the scale range
## (`geom_line()`).
```

```
## Warning: Removed 1 row containing missing values or values outside the scale range
## (`geom_point()`).
```

```
## Warning: Removed 1 row containing non-finite outside the scale range
## (`stat_bin()`).
```

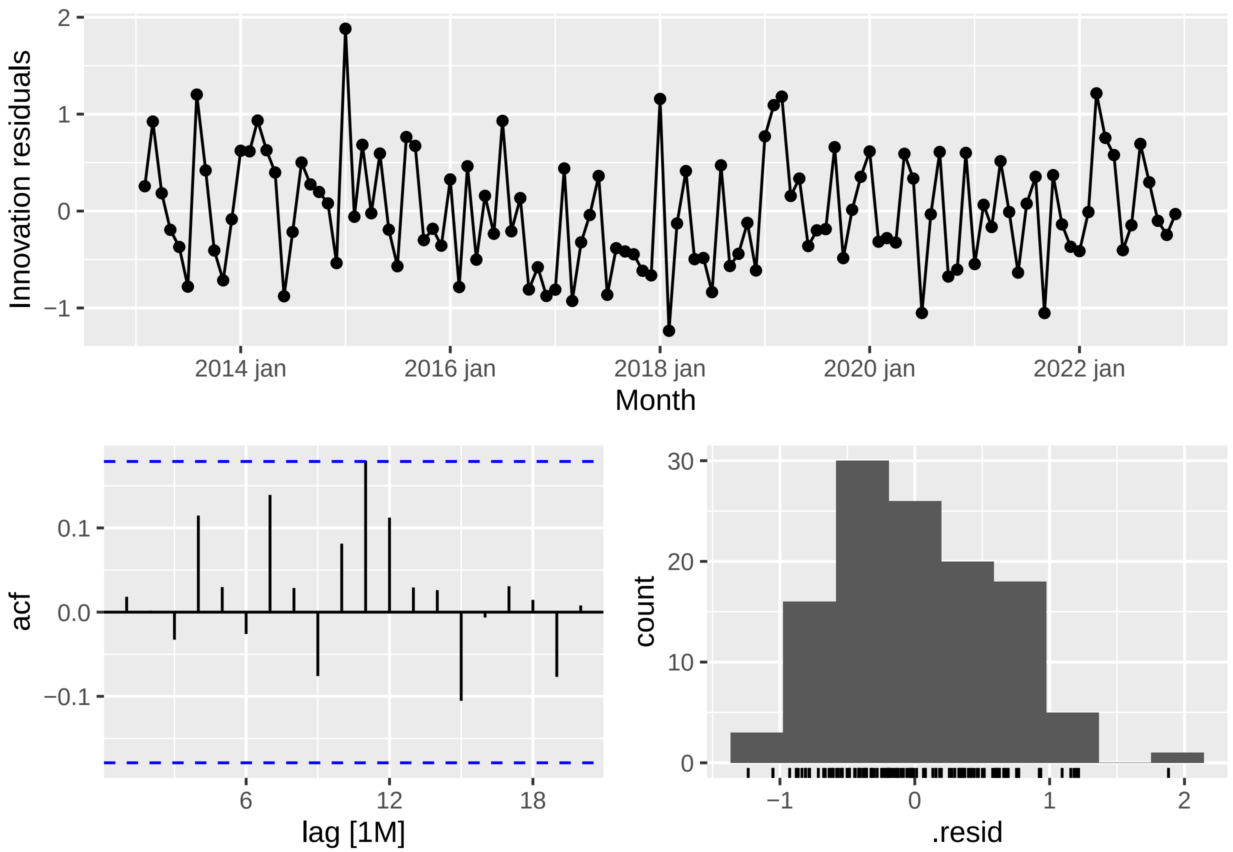


```
# report(reg_log1)

augment(reg_log5) |> features(.innov, ljung_box, lag=18, dof = 6)
```

```
## # A tibble: 1 x 3
##   .model lb_stat lb_pvalue
##   <chr>    <dbl>     <dbl>
## 1 arima     14.2     0.290
```

```
augment(reg_log5) |> features(.innov, ljung_box, lag=24, dof = 6)
```

```
## # A tibble: 1 x 3
##   .model lb_stat lb_pvalue
##   <chr>    <dbl>     <dbl>
## 1 arima     16.3     0.575
```

```
accuracy(reg_log5, measures = point_accuracy_measures)
```

```
## # A tibble: 1 x 10
##   .model .type       ME  RMSE   MAE   MPE  MAPE  MASE RMSSE  ACF1
##   <chr>  <chr>    <dbl> <dbl> <dbl> <dbl> <dbl> <dbl> <dbl> <dbl>
## 1 arima  Training  30.5  142.  42.9 -17.4  51.7 0.286 0.358 0.179
```

```
# diagnostic tests

# ts object

resid_model5 <- reg_log5 %>%
  residuals() %>%
  as_tibble()

resid_model5_ts <- ts(resid_model5[3], frequency = 12, start = c(2013,1))

# heteroskedasticity

FinTS::ArchTest(resid_model5_ts)
```

```
##
##  ARCH LM-test; Null hypothesis: no ARCH effects
##
## data:  resid_model5_ts
## Chi-squared = 7.2218, df = 12, p-value = 0.8426
```

```
# normality

# Shapiro-Wilk

shapiro.test(resid_model5_ts)
```

```
##
##  Shapiro-Wilk normality test
##
## data:  resid_model5_ts
## W = 0.98152, p-value = 0.1014
```

```
# manually calculating the SRMSE

residuos_modelo5_sem_NAs <- augment(reg_log5) %>% na.exclude()

desvpad5 <- sd(residuos_modelo5_sem_NAs$casosmais1_por_100k)

rmse5 <- sqrt(mean((residuos_modelo5_sem_NAs$casosmais1_por_100k - residuos_modelo5_sem_NAs$.fitted)^2))

print(rmse5)
```

```
## [1] 142.2017
```

```
# check it with the RMSE provided by 'accuracy' function above - OK

srmse5 <- rmse5/desvpad5

print(srmse5)
```

```
## [1] 0.4125431
```

model 6 (final model)

```
# Model 6 (final model): Controlling for contemporaneous precipitation and temperature, plus a two-peri

reg_log6 = base_dengue_pos2013_correc %>%
  as_tsibble() %>%
  model(arima = ARIMA( log(casosmais1_por_100k) ~
                      log(tmed) + log(lag(tmed, 1)) + log(lag(tmed, 2)) +
                      log(chuva) + log(lag(chuva, 1)) + log(lag(chuva, 2)) +
                       D1000,
                     greedy = FALSE,
                     stepwise = FALSE,
                     approximation = FALSE
                     )) %>%
  report(reg_log6)
```

```
## Series: casosmais1_por_100k
## Model: LM w/ ARIMA(2,0,4) errors
## Transformation: log(casosmais1_por_100k)
##
## Coefficients:
##          ar1      ar2      ma1     ma2     ma3     ma4  log(tmed)
##       1.6066  -0.8807  -0.2469  0.0904  0.0894  0.4858     4.2707
## s.e.  0.0528   0.0517   0.0961  0.0967  0.1021  0.1012     0.9194
##       log(lag(tmed, 1))  log(lag(tmed, 2))  log(chuva)  log(lag(chuva, 1))
##                  2.6180             2.5606      0.0547              0.0385
## s.e.             0.9008             0.9479      0.0648              0.0858
##       log(lag(chuva, 2))   D1000  intercept
##                   0.0551  0.2239   -27.6900
## s.e.              0.0636  0.2445     6.8879
##
## sigma^2 estimated as 0.3673:  log likelihood=-104.22
## AIC=238.44   AICc=243.06   BIC=280.26
```

```
reg_log6 |> gg_tsresiduals()
```

```
## Warning: Removed 2 rows containing missing values or values outside the scale range
## (`geom_line()`).
```

```
## Warning: Removed 2 rows containing missing values or values outside the scale range
## (`geom_point()`).
```

```
## Warning: Removed 2 rows containing non-finite outside the scale range
## (`stat_bin()`).
```

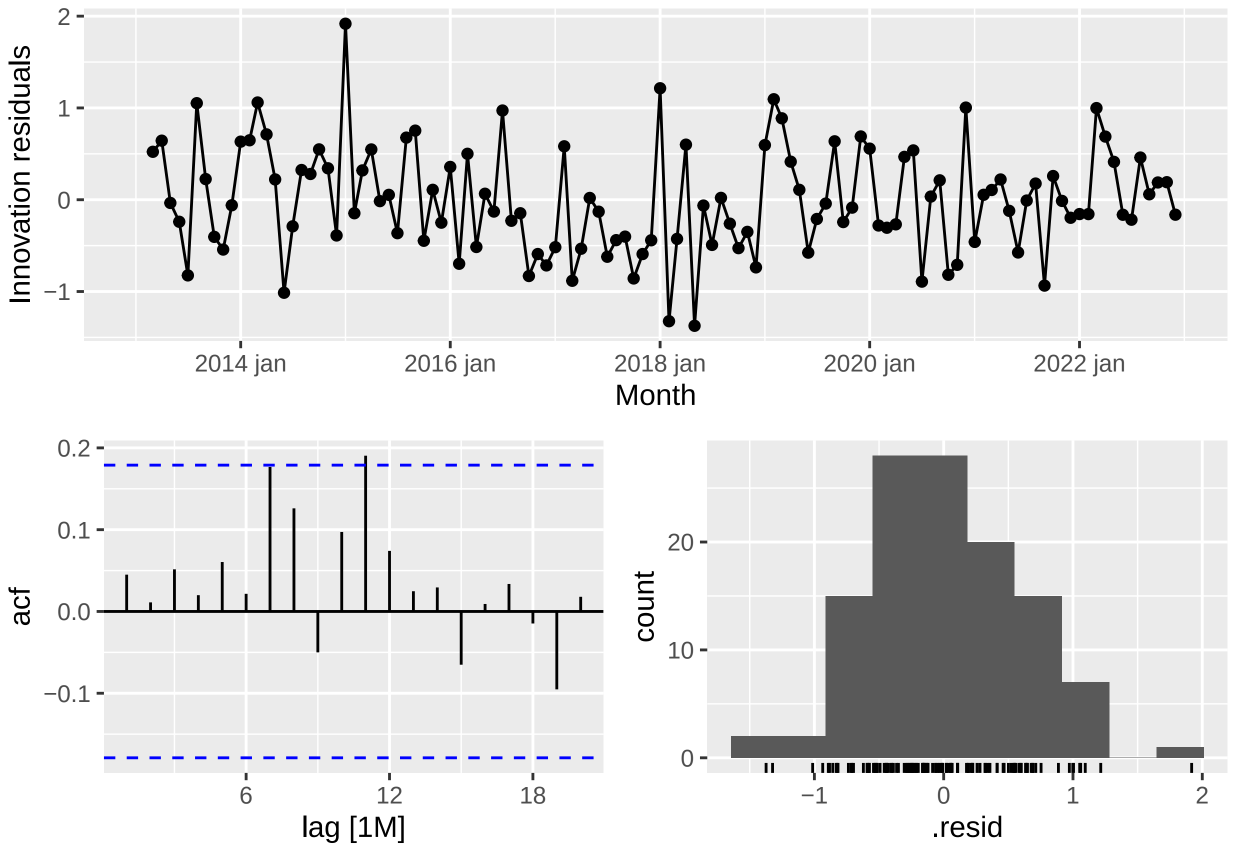


```
# report(reg_log1)

augment(reg_log6) |> features(.innov, ljung_box, lag=18, dof = 6)
```

```
## # A tibble: 1 x 3
##   .model lb_stat lb_pvalue
##   <chr>    <dbl>     <dbl>
## 1 arima     15.3     0.228
```

```
augment(reg_log6) |> features(.innov, ljung_box, lag=24, dof = 6)
```

```
## # A tibble: 1 x 3
##   .model lb_stat lb_pvalue
##   <chr>    <dbl>     <dbl>
## 1 arima     17.3     0.501
```

```
accuracy(reg_log6, measures = point_accuracy_measures)
```

```
## # A tibble: 1 x 10
##   .model .type       ME  RMSE   MAE   MPE  MAPE  MASE RMSSE  ACF1
##   <chr>  <chr>    <dbl> <dbl> <dbl> <dbl> <dbl> <dbl> <dbl> <dbl>
## 1 arima  Training  30.5  142.  43.7 -17.3  49.9 0.291 0.358 0.421
```

```r
# diagnostic tests

# ts object

resid_model6 <- reg_log6 %>%
  residuals() %>%
  as_tibble()

resid_model6_ts <- ts(resid_model6[3], frequency = 12, start = c(2013,1))

# heteroskedasticity

FinTS::ArchTest(resid_model6_ts)
```

```
##
##  ARCH LM-test; Null hypothesis: no ARCH effects
##
## data:  resid_model6_ts
## Chi-squared = 9.2423, df = 12, p-value = 0.6821
```

```r
# normality

# Shapiro-Wilk

shapiro.test(resid_model6_ts)
```

```
##
##  Shapiro-Wilk normality test
##
## data:  resid_model6_ts
## W = 0.98918, p-value = 0.4765
```

```r
# SRMSE

residuos_modelo6_sem_NAs <- augment(reg_log6) %>% na.exclude()

desvpad6 <- sd(residuos_modelo6_sem_NAs$casosmais1_por_100k)

rmse6 <- sqrt(mean((residuos_modelo6_sem_NAs$casosmais1_por_100k - residuos_modelo6_sem_NAs$.fitted)^2)

print(rmse6)
```

```
## [1] 142.1989
```

```r
# check it with the RMSE provided by 'accuracy' function above - OK

srmse6 <- rmse6/desvpad6

print(srmse6)
```

```
## [1] 0.4108621
```

appendix: additional models

```
# model with 3 lags
mod_3lags = base_dengue_pos2013_correc %>%
  as_tsibble() %>%
  model(arima = ARIMA( log(casosmais1_por_100k) ~
                      log(tmed) + log(lag(tmed, 1)) + log(lag(tmed, 2)) + log(lag(tmed, 3)) +
                      log(chuva) + log(lag(chuva, 1)) + log(lag(chuva, 2)) + log(lag(chuva, 3)) +
                       D1000,
                    greedy = FALSE,
                    stepwise = FALSE,
                    approximation = FALSE
                    )) %>%
  report(mod_3lags)
```

```
## Series: casosmais1_por_100k
## Model: LM w/ ARIMA(2,0,4) errors
## Transformation: log(casosmais1_por_100k)
##
## Coefficients:
##          ar1      ar2      ma1     ma2     ma3     ma4  log(tmed)
##       1.6012  -0.8840  -0.2627  0.0976  0.0955  0.5424     4.3442
## s.e.  0.0499   0.0494   0.0899  0.0954  0.1013  0.1009     0.9365
##       log(lag(tmed, 1))  log(lag(tmed, 2))  log(lag(tmed, 3))  log(chuva)
##                  2.5217             2.6274            -0.1590      0.0766
## s.e.             0.9757             1.0014             0.9478      0.0690
##       log(lag(chuva, 1))  log(lag(chuva, 2))  log(lag(chuva, 3))   D1000
##                   0.1251              0.1737              0.0889  0.2321
## s.e.              0.1051              0.1009              0.0614  0.2424
##       intercept
##        -28.6465
## s.e.     8.4895
##
## sigma^2 estimated as 0.366:  log likelihood=-102.74
## AIC=239.48   AICc=245.48   BIC=286.87
```

```
mod_3lags |> gg_tsresiduals()
```

```
## Warning: Removed 3 rows containing missing values or values outside the scale range
## (`geom_line()`).
```

```
## Warning: Removed 3 rows containing missing values or values outside the scale range
## (`geom_point()`).
```

```
## Warning: Removed 3 rows containing non-finite outside the scale range
## (`stat_bin()`).
```

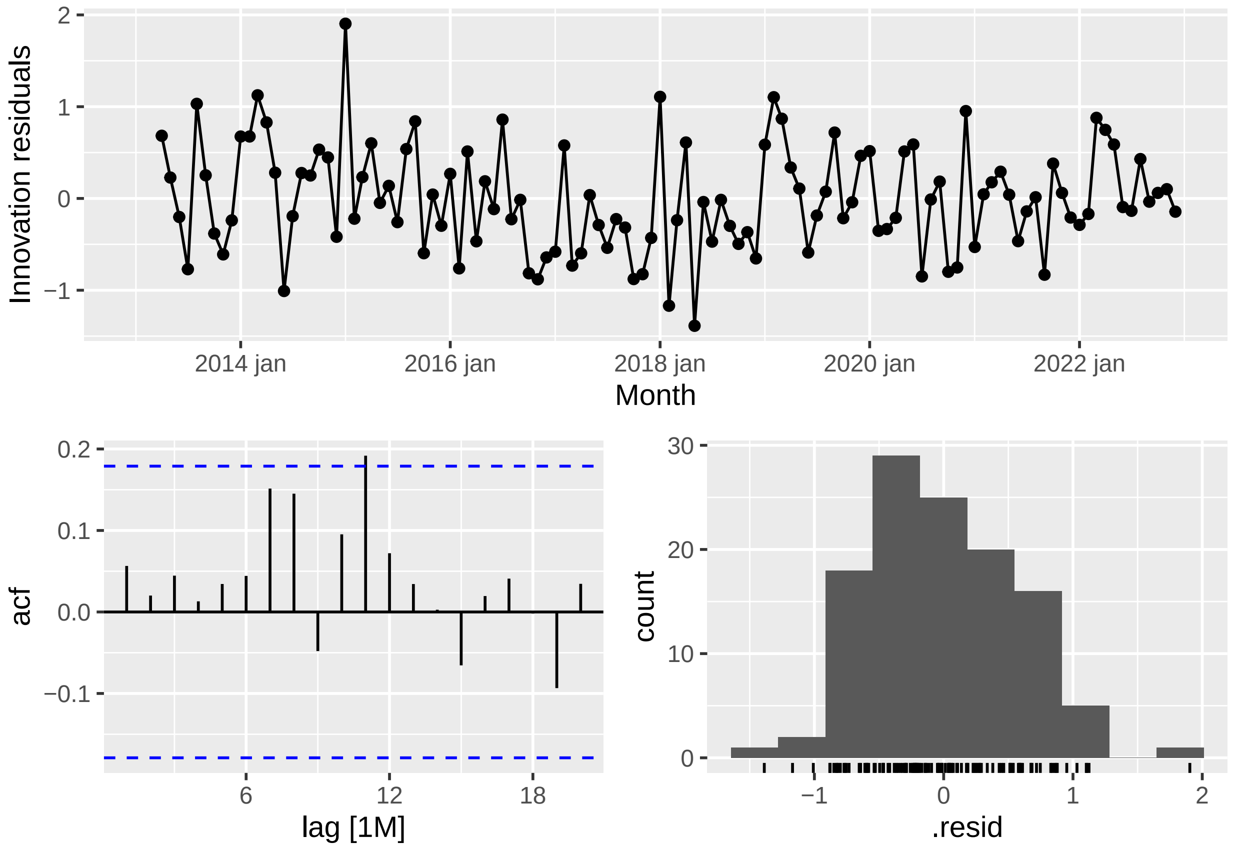


```
# report(reg_log1)

augment(mod_3lags) |> features(.innov, ljung_box, lag=18, dof = 6)
```

```
## # A tibble: 1 x 3
##   .model lb_stat lb_pvalue
##   <chr>    <dbl>     <dbl>
## 1 arima     14.7     0.258
```

```
augment(mod_3lags) |> features(.innov, ljung_box, lag=24, dof = 6)
```

```
## # A tibble: 1 x 3
##   .model lb_stat lb_pvalue
##   <chr>    <dbl>     <dbl>
## 1 arima     17.6     0.485
```

```
# models with temperature only

mod_temp1 = base_dengue_pos2013_correc %>%
  as_tsibble() %>%
  model(arima = ARIMA( log(casosmais1_por_100k) ~
                      log(tmed) + log(lag(tmed, 1)) +
                       D1000,
                    greedy = FALSE,
```

```
                      stepwise = FALSE,
                      approximation = FALSE
                      )) %>%
  report(mod_temp1)
```

```
## Series: casosmais1_por_100k
## Model: LM w/ ARIMA(2,0,0)(1,0,0)[12] errors
## Transformation: log(casosmais1_por_100k)
##
## Coefficients:
##          ar1      ar2    sar1  log(tmed)  log(lag(tmed, 1))   D1000  intercept
##       1.4735  -0.6649  0.2267      2.295             1.5239  0.2270    -9.5512
## s.e.  0.0801   0.0839  0.1176      0.898             0.9120  0.2652     4.7834
##
## sigma^2 estimated as 0.4079:  log likelihood=-114.1
## AIC=244.2   AICc=245.5   BIC=266.5
```

```
mod_temp1 |> gg_tsresiduals()
```

```
## Warning: Removed 1 row containing missing values or values outside the scale range
## (`geom_line()`).
```

```
## Warning: Removed 1 row containing missing values or values outside the scale range
## (`geom_point()`).
```

```
## Warning: Removed 1 row containing non-finite outside the scale range
## (`stat_bin()`).
```

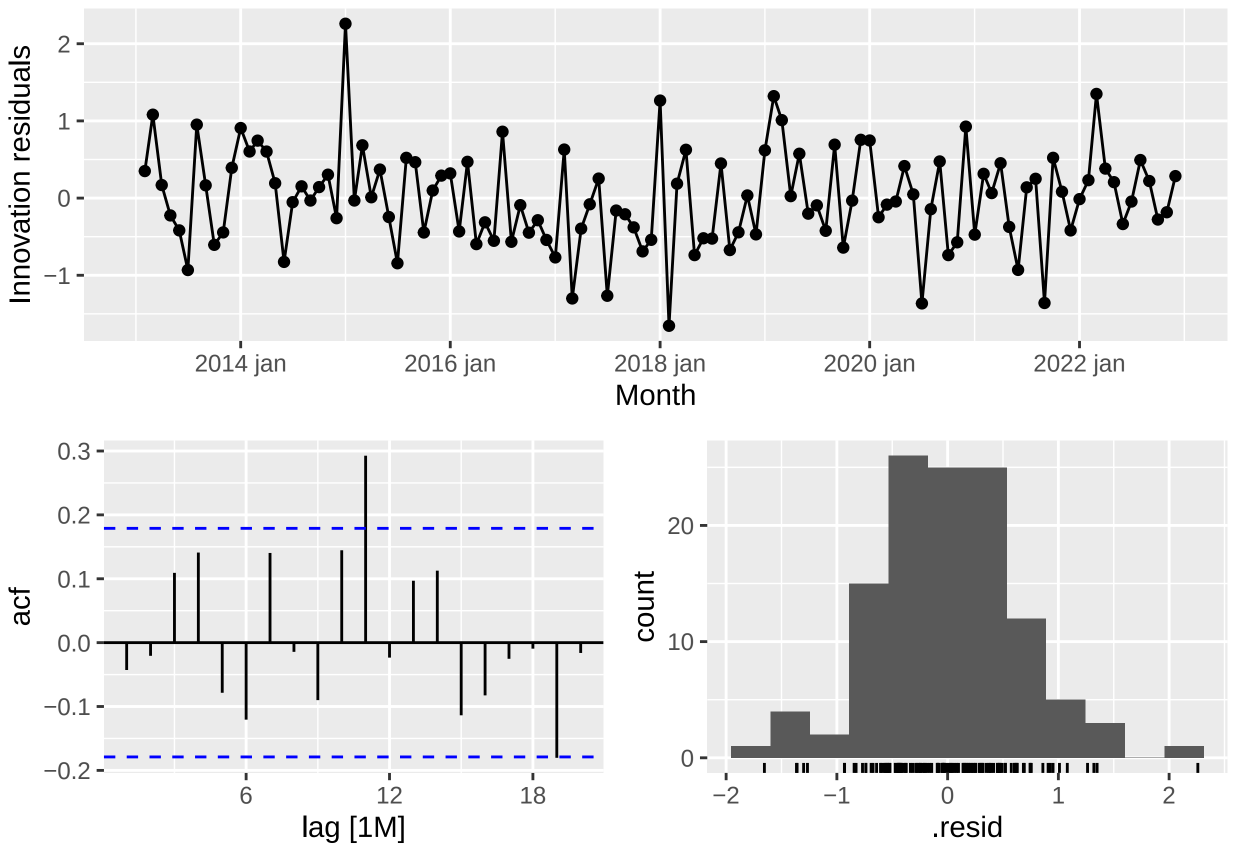


```
augment(mod_temp1) |> features(.innov, ljung_box, lag=18, dof = 3)
```

```
## # A tibble: 1 x 3
##   .model lb_stat lb_pvalue
##   <chr>    <dbl>     <dbl>
## 1 arima     30.6   0.00987
```

```
augment(mod_temp1) |> features(.innov, ljung_box, lag=24, dof = 3)
```

```
## # A tibble: 1 x 3
##   .model lb_stat lb_pvalue
##   <chr>    <dbl>     <dbl>
## 1 arima     35.8    0.0228
```

```
mod_temp2 = base_dengue_pos2013_correc %>%
  as_tsibble() %>%
  model(arima = ARIMA( log(casosmais1_por_100k) ~
                         log(tmed) + log(lag(tmed, 1)) + log(lag(tmed, 2)) +
                          D1000,
                       greedy = FALSE,
                       stepwise = FALSE,
                       approximation = FALSE
                       )) %>%
  report(mod_temp2)
```

```
## Series: casosmais1_por_100k
## Model: LM w/ ARIMA(2,0,0)(1,0,0)[12] errors
## Transformation: log(casosmais1_por_100k)
##
## Coefficients:
##          ar1      ar2    sar1  log(tmed)  log(lag(tmed, 1))  log(lag(tmed, 2))
##       1.4625  -0.6491  0.2208     2.9072             2.3064             1.4838
## s.e.  0.0815   0.0849  0.1132     0.9869             1.0467             0.9914
##        D1000  intercept
##       0.1729   -18.4932
## s.e.  0.2671     7.6724
##
## sigma^2 estimated as 0.4025:  log likelihood=-112.3
## AIC=242.6   AICc=244.24   BIC=267.69
```

```
mod_temp2 |> gg_tsresiduals()
```

```
## Warning: Removed 2 rows containing missing values or values outside the scale range
## (`geom_line()`).

## Warning: Removed 2 rows containing missing values or values outside the scale range
## (`geom_point()`).

## Warning: Removed 2 rows containing non-finite outside the scale range
## (`stat_bin()`).
```

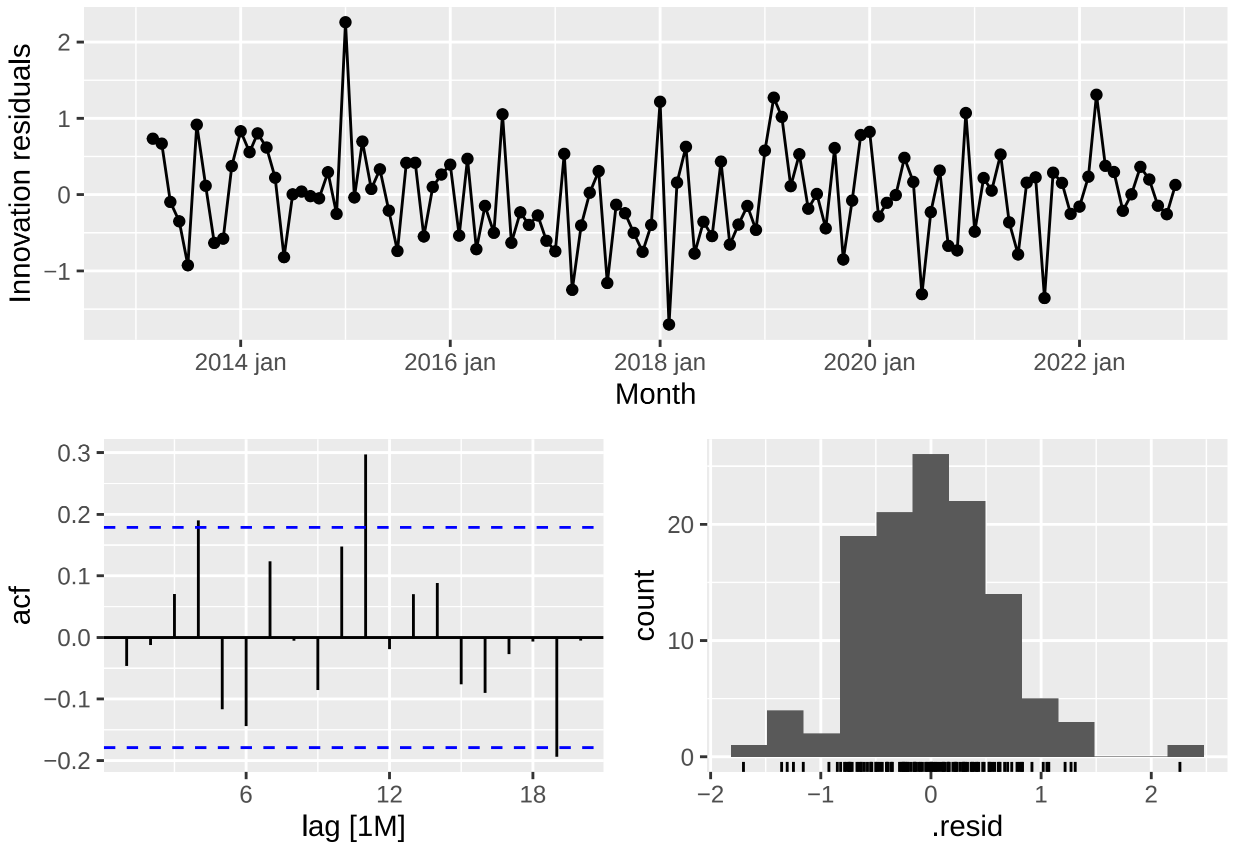

```r
augment(mod_temp2) |> features(.innov, ljung_box, lag=18, dof = 3)
```

```
## # A tibble: 1 x 3
##   .model lb_stat lb_pvalue
##   <chr>    <dbl>     <dbl>
## 1 arima     31.0   0.00892
```

```r
augment(mod_temp2) |> features(.innov, ljung_box, lag=24, dof = 3)
```

```
## # A tibble: 1 x 3
##   .model lb_stat lb_pvalue
##   <chr>    <dbl>     <dbl>
## 1 arima     37.0    0.0167
```

```r
# models with precipitation only

mod_precip1 = base_dengue_pos2013_correc %>%
  as_tsibble() %>%
  model(arima = ARIMA( log(casosmais1_por_100k) ~
                         log(chuva) + log(lag(chuva, 1)) +
                          D1000,
                       greedy = FALSE,
                       stepwise = FALSE,
                       approximation = FALSE
                       )) %>%
  report(mod_precip1)
```

```
## Series: casosmais1_por_100k
## Model: LM w/ ARIMA(2,0,0)(2,1,0)[12] errors
## Transformation: log(casosmais1_por_100k)
##
## Coefficients:
##          ar1      ar2     sar1     sar2  log(chuva)  log(lag(chuva, 1))   D1000
##       1.3393  -0.4592  -0.5897  -0.3783     -0.0620             -0.0551  0.2710
## s.e.  0.0968   0.0958   0.1054   0.0922      0.0478              0.0485  0.2736
##
## sigma^2 estimated as 0.4156:  log likelihood=-105.99
## AIC=227.99   AICc=229.44   BIC=249.45
```

```r
mod_precip1 |> gg_tsresiduals()
```

```
## Warning: Removed 1 row containing missing values or values outside the scale range
## (‘geom_line()‘).

## Warning: Removed 1 row containing missing values or values outside the scale range
## (‘geom_point()‘).

## Warning: Removed 1 row containing non-finite outside the scale range
## (‘stat_bin()‘).
```

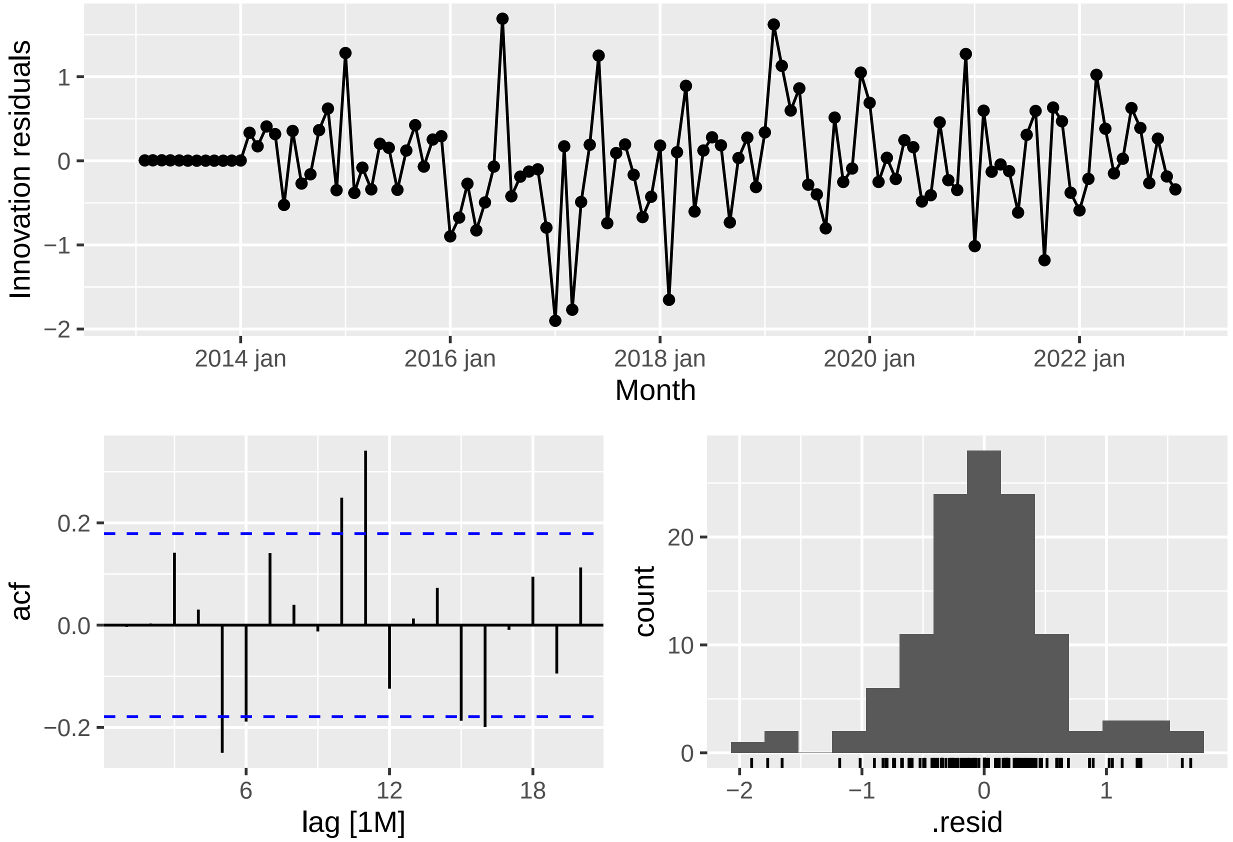


```
augment(mod_precip1) |> features(.innov, ljung_box, lag=18, dof = 6)
```

```
## # A tibble: 1 x 3
##   .model lb_stat   lb_pvalue
##   <chr>    <dbl>       <dbl>
## 1 arima     56.0 0.000000119
```

```
augment(mod_precip1) |> features(.innov, ljung_box, lag=24, dof = 6)
```

```
## # A tibble: 1 x 3
##   .model lb_stat   lb_pvalue
##   <chr>    <dbl>       <dbl>
## 1 arima     67.3 0.000000131
```

```
mod_precip2 = base_dengue_pos2013_correc %>%
  as_tsibble() %>%
  model(arima = ARIMA( log(casosmais1_por_100k) ~
                          log(chuva) + log(lag(chuva, 1)) + log(lag(chuva, 2)) +
                           D1000,
                        greedy = FALSE,
                        stepwise = FALSE,
                        approximation = FALSE
                        )) %>%
  report(mod_precip2)
```

```
## Series: casosmais1_por_100k
## Model: LM w/ ARIMA(0,0,5)(1,0,0)[12] errors
## Transformation: log(casosmais1_por_100k)
##
## Coefficients:
##          ma1     ma2     ma3     ma4     ma5    sar1  log(chuva)
##       1.3537  1.3734  1.1827  0.8247  0.4962  0.4267       0.028
## s.e.  0.0988  0.1691  0.2018  0.1524  0.1190  0.1124       0.060
##       log(lag(chuva, 1))  log(lag(chuva, 2))   D1000  intercept
##                   0.0525              0.0774  0.1377     1.7449
## s.e.              0.0776              0.0614  0.2659     0.9151
##
## sigma^2 estimated as 0.3995:  log likelihood=-111.3
## AIC=246.59   AICc=249.51   BIC=280.04
```

```
mod_precip2 |> gg_tsresiduals()
```

```
## Warning: Removed 2 rows containing missing values or values outside the scale range
## (`geom_line()`).

## Warning: Removed 2 rows containing missing values or values outside the scale range
## (`geom_point()`).

## Warning: Removed 2 rows containing non-finite outside the scale range
## (`stat_bin()`).
```

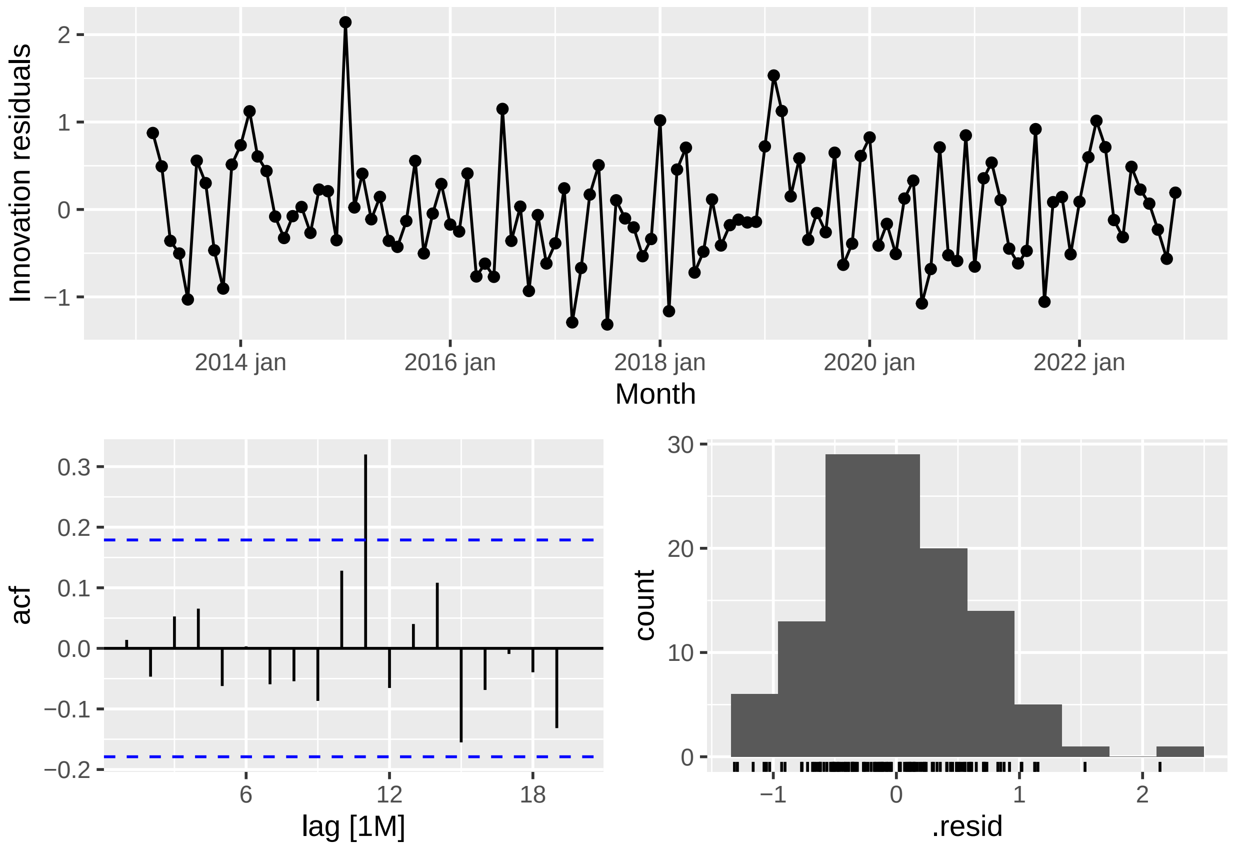

```
augment(mod_precip2) |> features(.innov, ljung_box, lag=18, dof = 6)
```

```
## # A tibble: 1 x 3
##   .model lb_stat lb_pvalue
##   <chr>    <dbl>     <dbl>
## 1 arima     25.7    0.0117
```

```
augment(mod_precip2) |> features(.innov, ljung_box, lag=24, dof = 6)
```

```
## # A tibble: 1 x 3
##   .model lb_stat lb_pvalue
##   <chr>    <dbl>     <dbl>
## 1 arima     29.0    0.0480
```

mes	ano	tmed	casos	casosmais1	chuva	pop	casosmais1_p00k
1	1998	26,1	237	238	107,0	930.681	25,572672
2	1998	25,6	331	332	283,0	931.757	35,631620
3	1998	25,4	562	563	219,0	932.832	60,353825
4	1998	22,9	187	188	71,0	933.908	20,130462
5	1998	19,2	32	33	111,0	934.984	3,529473
6	1998	17,9	11	12	20,0	936.059	1,2819700
7	1998	18,8	6	7	9,0	937.135	0,7469574
8	1998	21,6	4	5	12,0	938.211	0,5329293
9	1998	22	7	8	79,0	939.286	0,8517104
10	1998	21,8	7	8	167,0	940.362	0,8507362
11	1998	23,3	5	6	97,0	941.438	0,637323
12	1998	24,2	8	9	311,0	942.513	0,9548936
1	1999	25,2	7	8	386,0	943.589	0,8478267
2	1999	25,1	12	13	242,0	944.665	1,3761497
3	1999	25,1	27	28	149,0	945.740	2,9606435
4	1999	22,4	49	50	79,0	946.816	5,2808571
5	1999	19,1	8	9	45,0	947.892	0,9494755
6	1999	18,6	3	4	71,0	948.967	0,4215108
7	1999	20,1	1	2	2,0	950.043	0,2105167
8	1999	20,2	3	4	1,0	951.122	0,4205557
9	1999	21,5	3	4	76,0	952.202	0,4200790
10	1999	22,4	0	1	27,0	953.281	0,1049008
11	1999	22,6	0	1	80,0	954.361	0,1047821
12	1999	24,8	4	5	218,0	955.440	0,5233190
1	2000	25	6	7	251,0	956.520	0,7318198
2	2000	24,7	11	12	230,0	957.599	1,253134
3	2000	24,6	21	22	118,0	958.678	2,2948260
4	2000	23,2	15	16	4,0	959.758	1,6670873
5	2000	20,3	8	9	2,0	960.837	0,9366831
6	2000	19,6	4	5	8,0	961.917	0,5197955
7	2000	17,1	4	5	69,0	962.996	0,5192129
8	2000	20	3	4	68,0	964.661	0,4146534
9	2000	21,6	0	1	98,0	966.326	0,1034847
10	2000	25,2	2	3	127,0	967.991	0,3099201
11	2000	23,8	2	3	328,0	969.656	0,3093879
12	2000	24,1	5	6	301,0	971.321	0,6177151
1	2001	25,5	32	33	150,0	972.987	3,3916195
2	2001	25,9	38	39	284,0	974.652	4,0014299
3	2001	25,2	160	161	97,0	976.317	16,490551
4	2001	24,7	223	224	42,0	977.982	22,904312
5	2001	20,6	136	137	82,0	979.647	13,984631
6	2001	19,8	21	22	38,0	981.312	2,2418967
7	2001	19,1	13	14	22,0	982.977	1,4242449
8	2001	21,1	10	11	26,0	983.981	1,1179078
9	2001	21,7	2	3	69,0	984.985	0,3045732
10	2001	22,8	2	3	211,0	985.989	0,3042631
11	2001	24,5	5	6	109,0	986.993	0,6079072
12	2001	23,6	86	87	154,0	987.997	8,8056984
1	2002	24,7	224	225	312,0	989.001	22,750241
2	2002	23,9	364	365	163,0	990.004	36,868522
3	2002	25,7	348	349	152,0	991.008	35,216656
4	2002	24,7	266	267	28,0	992.012	26,914990
5	2002	21,2	156	157	73,0	993.016	15,810417
6	2002	20,9	50	51	1,0	994.020	5,1306810
7	2002	18,8	8	9	6,0	995.024	0,9045007
8	2002	22,4	9	10	71,0	996.015	1,0040007
9	2002	21,1	6	7	49,0	997.006	0,7021018
10	2002	26	4	5	63,0	997.998	0,5010032

11	2002	23,8	7	8	189,0	998.989	0,8008098
12	2002	25,2	22	23	181,0	999.980	2,3000463
1	2003	24,7	90	91	323,0	1.000.971	9,0911724
2	2003	26,1	91	92	196,0	1.001.962	9,1819834
3	2003	24,1	125	126	93,0	1.002.953	12,562897
4	2003	22,4	76	77	48,0	1.003.945	7,6697466
5	2003	18,7	28	29	41,0	1.004.936	2,8857568
6	2003	19,8	7	8	17,0	1.005.927	0,795286
7	2003	18,6	2	3	15,0	1.006.918	0,2979388
8	2003	18,6	0	1	17,0	1.008.999	0,0991081
9	2003	21,8	0	1	21,0	1.011.080	0,0989041
10	2003	23,2	1	2	90,0	1.013.160	0,1974021
11	2003	23,3	0	1	174,0	1.015.241	0,098498
12	2003	24,6	3	4	246,0	1.017.322	0,3931892
1	2004	23,7	9	10	242,0	1.019.403	0,9809667
2	2004	23,7	8	9	189,0	1.021.483	0,8810717
3	2004	23,2	6	7	59,0	1.023.564	0,6838849
4	2004	22,8	3	4	66,0	1.025.645	0,3899985
5	2004	18,3	1	2	76,0	1.027.726	0,1946044
6	2004	18	0	1	62,0	1.029.806	0,0971056
7	2004	17,7	0	1	91,0	1.031.887	0,0969098
8	2004	19,4	0	1	1,0	1.033.039	0,0968018
9	2004	24,2	0	1	11,0	1.034.190	0,0966940
10	2004	21,5	0	1	188,0	1.035.342	0,0965864
11	2004	23,2	0	1	130,0	1.036.493	0,0964791
12	2004	23,8	3	4	221,0	1.037.645	0,3854883
1	2005	24,2	5	6	360,0	1.038.797	0,5775914
2	2005	24,7	7	8	45,0	1.039.948	0,7692691
3	2005	24,6	8	9	269,0	1.041.100	0,8644705
4	2005	24	38	39	27,0	1.042.251	3,7419000
5	2005	20,9	29	30	160,0	1.043.403	2,8752078
6	2005	19,9	17	18	41,0	1.044.554	1,7232228
7	2005	18,3	8	9	3,0	1.045.706	0,8606625
8	2005	20,9	2	3	7,0	1.046.849	0,2865743
9	2005	21,1	2	3	44,0	1.047.992	0,2862618
10	2005	24,6	0	1	168,0	1.049.135	0,0953166
11	2005	23,7	0	1	37,0	1.050.277	0,0952129
12	2005	23,9	3	4	135,0	1.051.420	0,3804378
1	2006	25,7	8	9	254,0	1.052.563	0,8550557
2	2006	25	24	25	219,0	1.053.706	2,3725786
3	2006	25,1	187	188	235,0	1.054.849	17,822461
4	2006	22,3	292	293	23,0	1.055.992	27,746435
5	2006	18,4	129	130	3,0	1.057.134	12,297396
6	2006	18,4	31	32	18,0	1.058.277	3,0237825
7	2006	18,6	7	8	41,0	1.059.420	0,7551301
8	2006	20,4	16	17	21,0	1.057.743	1,6071955
9	2006	20,8	10	11	58,0	1.056.066	1,0416014
10	2006	23,2	17	18	92,0	1.054.389	1,7071494
11	2006	23,4	10	11	241,0	1.052.712	1,0449198
12	2006	24,6	11	12	185,0	1.051.035	1,1417312
1	2007	23,6	169	170	402,0	1.049.359	16,200373
2	2007	25,1	922	923	168,0	1.047.682	88,099286
3	2007	24,9	3213	3214	142,0	1.046.005	307,26440
4	2007	23,4	4207	4208	73,0	1.044.328	402,93863
5	2007	19	2364	2365	71,0	1.042.651	226,82569
6	2007	18,9	300	301	31,0	1.040.974	28,915229
7	2007	17,6	67	68	191,0	1.039.297	6,5428842
8	2007	20,2	17	18	1,0	1.040.743	1,7295343
9	2007	23	35	36	2,0	1.042.188	3,4542706
10	2007	24,4	49	50	87,0	1.043.634	4,7909527

11	2007	22,8	57	58	170,0	1.045.079	5,5498179
12	2007	24,3	42	43	118,0	1.046.525	4,1088367
1	2008	23,5	40	41	282,0	1.047.971	3,9123238
2	2008	24	37	38	281,0	1.049.416	3,6210613
3	2008	23,3	72	73	169,0	1.050.862	6,9466802
4	2008	21,9	79	80	124,0	1.052.307	7,6023423
5	2008	18,5	21	22	63,0	1.053.753	2,0877761
6	2008	18,7	10	11	73,0	1.055.198	1,0424579
7	2008	18,5	8	9	1,0	1.056.644	0,8517532
8	2008	20,7	9	10	70,0	1.057.313	0,9457939
9	2008	20,6	2	3	38,0	1.057.982	0,2835588
10	2008	24,2	8	9	90,0	1.058.650	0,8501391
11	2008	24,3	6	7	88,0	1.059.319	0,6608018
12	2008	24,9	14	15	252,0	1.059.988	1,4151106
1	2009	24,8	17	18	326,0	1.060.657	1,697062
2	2009	25,9	29	30	283,0	1.061.325	2,8266546
3	2009	26,1	53	54	118,0	1.061.994	5,084774
4	2009	23,3	40	41	26,0	1.062.663	3,8582325
5	2009	21,8	25	26	55,0	1.063.332	2,4451452
6	2009	16,7	16	17	64,0	1.064.000	1,5977439
7	2009	17,9	2	3	75,0	1.064.669	0,2817777
8	2009	19,9	3	4	54,0	1.066.030	0,375224
9	2009	22,3	2	3	114,0	1.067.391	0,2810592
10	2009	23,5	3	4	83,0	1.068.752	0,3742684
11	2009	26,1	3	4	271,0	1.070.112	0,3737925
12	2009	25,3	7	8	362,0	1.071.473	0,746635
1	2010	25,8	65	66	318,0	1.072.834	6,1519303
2	2010	26,8	249	250	59,0	1.074.195	23,273245
3	2010	25,4	626	627	182,0	1.075.556	58,295448
4	2010	23,4	942	943	57,0	1.076.917	87,564820
5	2010	20,3	630	631	34,0	1.078.277	58,519267
6	2010	18,9	84	85	24,0	1.079.638	7,8730080
7	2010	20,6	11	12	57,0	1.080.999	1,1100842
8	2010	20,3	10	11	1,0	1.081.781	1,0168414
9	2010	23	5	6	74,0	1.082.564	0,5542400
10	2010	22,9	5	6	75,0	1.083.346	0,5538398
11	2010	24,7	8	9	193,0	1.084.128	0,8301602
12	2010	26,2	12	13	215,0	1.084.910	1,19825[illegible]
1	2011	26,5	68	69	404,0	1.085.693	6,3553906
2	2011	26,9	288	289	142,0	1.086.475	26,599789
3	2011	24,6	658	659	214,0	1.087.257	60,611244
4	2011	23,9	1202	1203	100,0	1.088.039	110,56586
5	2011	20,2	714	715	25,0	1.088.822	65,667329
6	2011	17,8	133	134	45,0	1.089.604	12,298048
7	2011	20,1	26	27	6,0	1.090.386	2,4761873
8	2011	21,6	11	12	28,0	1.091.073	1,099834
9	2011	22,9	13	14	13,0	1.091.760	1,2823331
10	2011	24,1	23	24	153,0	1.092.447	2,1969029
11	2011	23,7	22	23	209,0	1.093.134	2,1040421
12	2011	25,2	20	21	227,0	1.093.821	1,9198753
1	2012	24,3	52	53	330,0	1.094.508	4,8423583
2	2012	26,9	56	57	113,0	1.095.195	5,2045526
3	2012	25,4	159	160	20,0	1.095.882	14,600112
4	2012	24	359	360	163,0	1.096.569	32,829671
5	2012	20,1	208	209	64,0	1.097.256	19,047514
6	2012	19,3	90	91	135,0	1.097.943	8,2882262
7	2012	18,4	18	19	42,0	1.098.630	1,729426
8	2012	20,7	12	13	1,0	1.102.483	1,1791568
9	2012	22,8	9	10	35,0	1.106.335	0,9038850
10	2012	25,5	7	8	89,0	1.110.188	0,7205986

11	2012	24,7	11	12	65,0	1.114.041	1,077159
12	2012	27	30	31	152,0	1.117.893	2,7730731
1	2013	24,4	143	144	216,0	1.121.746	12,837130
2	2013	25,5	496	497	120,0	1.125.599	44,154281
3	2013	24,3	1914	1915	241,0	1.129.451	169,55135
4	2013	22,1	2708	2709	96,0	1.133.304	239,03559
5	2013	20,2	1279	1280	66,0	1.137.157	112,56144
6	2013	19,5	302	303	54,0	1.141.009	26,555435
7	2013	19,5	35	36	74,0	1.144.862	3,1444837
8	2013	19,5	25	26	5,0	1.145.675	2,2694046
9	2013	21,6	35	36	38,0	1.146.488	3,140024
10	2013	22,5	29	30	96,0	1.147.301	2,614833
11	2013	23,8	21	22	78,0	1.148.114	1,916186
12	2013	25,5	43	44	98,0	1.148.927	3,8296615
1	2014	26,6	257	258	152,0	1.149.740	22,439865
2	2014	27,2	1655	1656	61,0	1.150.552	143,9308
3	2014	24,8	7638	7639	80,0	1.151.365	663,47316
4	2014	23	20623	20624	61,0	1.152.178	1790,0008
5	2014	20,3	10488	10489	26,0	1.152.991	909,72075
6	2014	20,2	1340	1341	6,0	1.153.804	116,22423
7	2014	18,9	146	147	25,0	1.154.617	12,731494
8	2014	20,7	51	52	12,0	1.155.407	4,5005782
9	2014	23	37	38	67,0	1.156.197	3,2866366
10	2014	24,6	39	40	35,0	1.156.987	3,4572550
11	2014	24,5	57	58	120,0	1.157.777	5,0095988
12	2014	24,9	74	75	270,0	1.158.567	6,4735119
1	2015	26,6	1466	1467	136,0	1.159.358	126,53560
2	2015	24,7	6919	6920	208,0	1.160.148	596,47583
3	2015	23,4	24745	24746	243,0	1.160.938	2131,5528
4	2015	22,8	23238	23239	24,0	1.161.728	2000,382
5	2015	19,8	7869	7870	82,0	1.162.518	676,97886
6	2015	19,3	1166	1167	22,0	1.163.308	100,3173
7	2015	19	85	86	33,0	1.164.098	7,3876941
8	2015	21	33	34	26,0	1.164.871	2,9187789
9	2015	23	42	43	148,0	1.165.643	3,6889500
10	2015	24,9	41	42	68,0	1.166.416	3,6007736
11	2015	24,3	52	53	171,0	1.167.189	4,5408254
12	2015	25,1	98	99	321,0	1.167.961	8,4763080
1	2016	24,7	399	400	236,0	1.168.734	34,225067
2	2016	25,9	625	626	283,0	1.169.507	53,526843
3	2016	24,6	1190	1191	205,0	1.170.279	101,77057
4	2016	24,7	701	702	13,0	1.171.052	59,946099
5	2016	19,5	184	185	84,0	1.171.825	15,787344
6	2016	17,4	26	27	180,0	1.172.597	2,302580
7	2016	19,1	25	26	2,0	1.173.370	2,2158398
8	2016	20,4	24	25	37,0	1.174.125	2,1292453
9	2016	21,4	30	31	30,0	1.174.880	2,6385677
10	2016	23,3	28	29	114,0	1.175.635	2,4667525
11	2016	23,3	26	27	121,0	1.176.390	2,2951578
12	2016	24,8	22	23	127,0	1.177.145	1,953880
1	2017	24,8	16	17	356,0	1.177.900	1,4432470
2	2017	25,7	37	38	102,0	1.178.654	3,2240154
3	2017	24	23	24	240,0	1.179.409	2,0349169
4	2017	22,7	12	13	88,0	1.180.164	1,1015415
5	2017	20,4	9	10	114,0	1.180.919	0,846798
6	2017	19	12	13	27,0	1.181.674	1,1001341
7	2017	18,1	6	7	1,0	1.182.429	0,5920017
8	2017	19,7	6	7	34,0	1.183.401	0,5915154
9	2017	23,7	13	14	13,0	1.184.373	1,1820598
10	2017	23,5	17	18	103,0	1.185.345	1,5185449

11	2017	23,1	11	12	234,0	1.186.317	1,0115337
12	2017	24,7	8	9	173,0	1.187.289	0,7580291
1	2018	24,5	39	40	205,0	1.188.262	3,3662623
2	2018	24,2	27	28	88,9	1.189.234	2,3544575
3	2018	25,9	33	34	81,3	1.190.206	2,8566491
4	2018	23,3	70	71	44,5	1.191.178	5,9604874
5	2018	20,9	45	46	8,6	1.192.150	3,8585753
6	2018	20,7	26	27	10,2	1.193.122	2,2629707
7	2018	20,5	11	12	10,2	1.194.094	1,0049460
8	2018	19,1	12	13	75,9	1.194.926	1,0879338
9	2018	22	12	13	50,5	1.195.757	1,087177
10	2018	23,1	12	13	142,5	1.196.589	1,0864217
11	2018	23,6	15	16	299,7	1.197.420	1,3362058
12	2018	25,4	16	17	69,8	1.198.252	1,4187333
1	2019	26,7	72	73	192,5	1.199.084	6,087983
2	2019	24,9	497	498	174,0	1.199.915	41,502936
3	2019	24,7	3791	3792	59,9	1.200.747	315,80[illegible]
4	2019	23,9	9346	9347	110,2	1.201.578	777,89357
5	2019	21,6	9383	9384	43,4	1.202.410	780,43273
6	2019	20	2603	2604	10,2	1.203.241	216,41542
7	2019	18,5	411	412	48,8	1.204.073	34,217194
8	2019	19,9	77	78	5,3	1.204.883	6,4736580
9	2019	23,2	65	66	50,0	1.205.693	5,4740310
10	2019	25,2	33	34	55,6	1.206.503	2,8180623
11	2019	24,3	21	22	160,3	1.207.313	1,8222288
12	2019	24,6	42	43	147,3	1.208.123	3,559241
1	2020	25,1	244	245	274,5	1.208.933	20,265813
2	2020	24,1	645	646	191,0	1.209.742	53,399797
3	2020	23,8	1048	1049	148,0	1.210.552	86,654659
4	2020	22,2	884	885	61,9	1.211.362	73,058244
5	2020	19	645	646	56,3	1.212.172	53,292759
6	2020	18,3	340	341	43,1	1.212.982	28,112533
7	2020	19,8	53	54	33,8	1.213.792	4,4488676
8	2020	19,9	13	14	24,4	1.214.579	1,1526626
9	2020	24,8	21	22	22,9	1.215.366	1,8101540
10	2020	25	16	17	44,2	1.216.153	1,3978501
11	2020	23,8	8	9	106,9	1.216.940	0,7395596
12	2020	24,9	32	33	187,5	1.217.727	2,7099660
1	2021	25,6	92	93	178,1	1.218.515	7,6322440
2	2021	24,6	253	254	258,8	1.219.302	20,831597
3	2021	24,8	510	511	81,3	1.220.089	41,8822[illegible]
4	2021	21,7	800	801	12,2	1.220.876	65,608641
5	2021	20	487	488	23,4	1.221.663	39,945555
6	2021	19,1	102	103	27,1	1.222.450	8,4257030
7	2021	17,4	24	25	33,8	1.223.237	2,0437576
8	2021	20,9	21	22	15,5	1.218.285	1,8058177
9	2021	24,7	11	12	6,6	1.213.332	0,9890118
10	2021	22,7	11	12	121,4	1.208.380	0,9930651
11	2021	23,6	16	17	83,8	1.203.428	1,4126317
12	2021	24,2	30	31	168,1	1.198.475	2,5866199
1	2022	24,5	57	58	312,9	1.193.523	4,8595633
2	2022	24,8	145	146	168,4	1.188.571	12,283663
3	2022	25,6	1167	1168	130,8	1.183.618	98,680471
4	2022	23,3	4244	4245	40,1	1.178.666	360,15297
5	2022	19	3757	3758	39,4	1.173.713	320,18035
6	2022	18,9	1057	1058	15,7	1.168.761	90,523203
7	2022	20,6	363	364	8,9	1.163.809	31,276616
8	2022	19,5	200	201	28,2	1.158.856	17,34468
9	2022	20	102	103	89,7	1.153.904	8,9262187
10	2022	23,1	73	74	120,4	1.148.952	6,4406536

11	2022	22,1	45	46	142,5	1.143.999	4,0209812
12	2022	23,5	50	51	324,1	1.139.047	4,4774271
